# Immune checkpoint inhibitors in cancer: the increased risk of atherosclerotic cardiovascular disease events and progression of coronary artery calcium

**DOI:** 10.1186/s12916-024-03261-x

**Published:** 2024-01-31

**Authors:** Bingxin Gong, Yusheng Guo, Yi Li, Jing Wang, Guofeng Zhou, Yong-hao Chen, Tong Nie, Ming Yang, Kun Luo, Chuansheng Zheng, Feng Pan, Bo Liang, Lian Yang

**Affiliations:** 1 Department of Radiology, Union Hospital, Tongji Medical College, Huazhong University of Science and Technology, Wuhan, 430022 China; 2 Hubei Key Laboratory of Molecular Imaging, Wuhan, 430022 China; 3 Department of Cardiology, State Key Laboratory of Complex Severe and Rare Diseases, Peking Union Medical College Hospital, Chinese Academy of Medical Sciences and Peking Union Medical College, Beijing, 100005 China

**Keywords:** Atherosclerosis, Immunotherapy, Cardiovascular disease, Coronary artery calcium, Imaging

## Abstract

**Background:**

Immune checkpoint inhibitors (ICIs) have contributed to a significant advancement in the treatment of cancer, leading to improved clinical outcomes in many individuals with advanced disease. Both preclinical and clinical investigations have shown that ICIs are associated with atherosclerosis and other cardiovascular events; however, the exact mechanism underlying this relationship has not been clarified.

**Methods:**

Patients diagnosed with stages III or IV non-small cell lung cancer (NSCLC) at the Wuhan Union Hospital from March 1, 2020, to April 30, 2022, were included in this retrospective study. Coronary artery calcium (CAC) volume and score were assessed in a subset of patients during non-ECG-gated chest CT scans at baseline and 3, 6, and 12 months after treatment. Propensity score matching (PSM) was performed in a 1:1 ratio to balance the baseline characteristics between the two groups.

**Results:**

Overall, 1458 patients (487 with ICI therapy and 971 without ICI therapy) were enrolled in this cardiovascular cohort study. After PSM, 446 patients were included in each group. During the entire period of follow-up (median follow-up 23.1 months), 24 atherosclerotic cardiovascular disease (ASCVD) events (4.9%) occurred in the ICI group, and 14 ASCVD events (1.4%) in the non-ICI group, before PSM; 24 ASCVD events (5.4%) occurred in the ICI group and 5 ASCVD events (1.1%) in the non-ICI group after PSM. The CAC imaging study group comprised 113 patients with ICI therapy and 133 patients without ICI therapy. After PSM, each group consisted of 75 patients. In the ICI group, the CAC volume/score increased from 93.4 mm^3^/96.9 (baseline) to 125.1 mm^3^/132.8 (at 12 months). In the non-ICI group, the CAC volume/score was increased from 70.1 mm^3^/68.8 (baseline) to 84.4 mm^3^/87.9 (at 12 months). After PSM, the CAC volume/score was increased from 85.1 mm^3^/76.4 (baseline) to 111.8 mm^3^/121.1 (12 months) in the ICI group and was increased from 74.9 mm^3^/76.8 (baseline) to 109.3 mm^3^/98.7 (12 months) in the non-ICI group. Both cardiovascular events and CAC progression were increased after the initiation of ICIs.

**Conclusions:**

Treatment with ICIs was associated with a higher rate of ASCVD events and a noticeable increase in CAC progression.

**Supplementary Information:**

The online version contains supplementary material available at 10.1186/s12916-024-03261-x.

## Background

The field of tumor treatment has reached a significant milestone with the introduction of immune checkpoint inhibitors (ICIs) targeting programmed cell death 1 (PD-1), programmed cell death-ligand 1 (PD-L1), and cytotoxic T-lymphocyte-associated protein 4 (CTLA-4) [1]. ICI monotherapy or ICI combined with other antineoplastic therapy has become the standard therapy for several advanced cancers, significantly improving the prognosis of patients [2, 3]. For example, in recent phase III studies, the use of pembrolizumab plus pemetrexed and platinum demonstrated impressive efficacy in patients with non-small cell lung cancer (NSCLC) [4]. Moreover, camrelizumab plus rivoceranib produced clinically significant improvement in patients with unresectable hepatocellular carcinoma [5].

With the widespread use of ICIs, immune-related adverse events (irAEs) such as endocrine dysfunction, diarrhea, and aspartate transaminase (AST) increase and various cardiovascular events have followed [6, 7]. Previously, atherosclerosis was generally not considered an irAE. However, in the past few years, cellular and animal research has revealed the potential roles of various immune checkpoint pathways in the activation, progression, and exacerbation of atherosclerosis [8–12]. ICIs regulate T-cell activation by targeting PD-1, PD-L1, and CTLA-4 to enhance their anti-tumor capacities. However, they also increase T-cell infiltration within atherosclerotic plaques and promote atherosclerosis [13]. In addition, several retrospective clinical studies have shown a non-negligible association between ICIs with atherosclerotic plaque and related cardiovascular events. For example, Drobni et al. [14] found that the risk of myocardial infarction, coronary revascularization, and ischemic stroke increased at varying degrees after the use of ICIs, and that total aortic plaque volume also showed a simultaneous increase. Similar findings were found in the melanoma patient cohort studied by Wang et al. [15]. In the study by Schiffer et al. [16], patients treated with ICIs who had cardiovascular events had worse baseline coronary and aortic calcification.

Coronary artery calcium (CAC) is now recognized as a valuable imaging biomarker that can offer direct proof of coronary atherosclerosis, which is the specific manifestation of systemic atherosclerosis in coronary arteries and is closely associated with a higher risk of atherosclerotic cardiovascular disease (ASCVD) and related cardiovascular events [17–20]. The JCCT-ICOS guidelines [21] state that CAC scores can be easily obtained from CT images for lung cancer screening to help predict cardiovascular disease events and provide essential information for cardiovascular disease risk stratification. Although a non-ECG-gated chest CT scan is not the gold standard for the assessment of CAC, it has been shown to be reliable, and its results are comparable to those obtained from ECG-gated CTA [22–24].

Therefore, we conducted a retrospective analysis of the medical files of patients diagnosed with advanced NSCLC and followed them up, aiming to evaluate whether the incidence of ASCVD events was increased following the start of ICI therapy. Simultaneously, we obtained CAC data for some patients from serial chest CT scans to further analyze the association between ICIs and atherosclerosis, without involving additional radiation exposure.

## Methods

This retrospective cohort study received approval from the Ethics Committee of Tongji Medical College (Institutional Review Board No. S054). The requirement for written informed consent was waived by the institutional review board.

### Study design and patients selection

This retrospective analysis was performed on consecutive patients with stages III or IV NSCLC at Wuhan Union Hospital from March 1, 2020, to April 30, 2022. Patients treated with ICIs therapy were defined as cases, and those treated with other antineoplastic therapies were defined as controls.

The inclusion and exclusion criteria of patients in our study were as follows. Inclusion criteria are as follows: (1) diagnosis of stages III or IV NSCLC according to the NCCN Clinical Practice Guidelines in Oncology: Non-Small Cell Lung Cancer (Version 3.2022) [25], (2) histologically or cytologically confirmed NSCLC, (3) age > 18 years, and (4) treatment with ICIs or other antineoplastic therapy for at least four consecutive cycles. Exclusion criteria are as follows: (1) combination with other primary malignant tumors, (2) NSCLC treated surgically, (3) then subsequently treated with ICIs (not when treatment started), and (4) incomplete medical records.

**Fig. 1:**
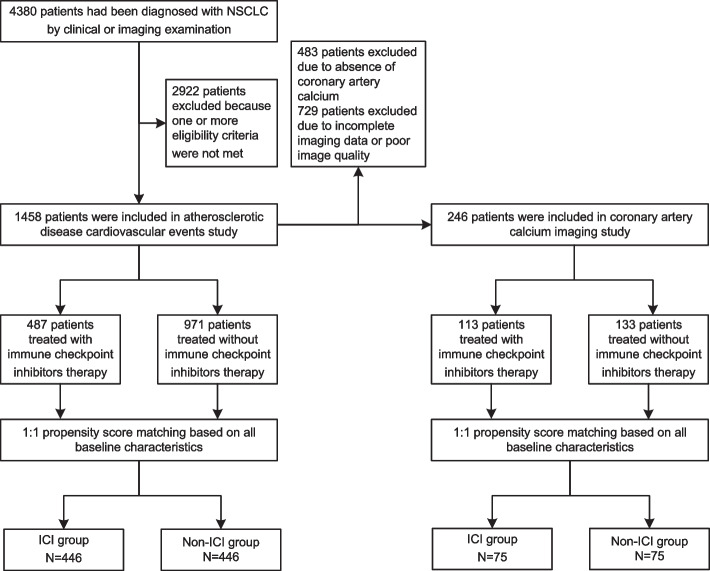
Flow diagram. Abbreviations: NSCLC, non-small cell lung cancer; ICI, immune checkpoint inhibitor

In addition, we conducted further screening to include patients with coronary artery calcified plaques before treatment and those who had undergone chest CT scans at 3 months (± 30 days), 6 months (± 30 days), and 12 months (± 30 days) after treatment into the imaging study. Ultimately, we included 1458 patients in the cardiovascular study, 246 of which were further enrolled in the CAC imaging study. After PSM, the two studies included 892 and 150 patients, respectively (Fig. 1).

### Procedures

Patient characteristics at baseline were collected from medical records and included sex, age, and body mass index, cancer characteristics (pathological types and stages), cardiovascular risk factors (hypertension, diabetes, smoking index, hyperlipidemia, history of cardiovascular disease, medications and chest radiation therapy), serum biochemical indices, ICI types (PD-1, PD-L1, and CTLA-4 antibodies), and cycles of use and survival information. In addition, we collected information regarding genetic mutations (EGFR, ALK, and KRAS).

### Coronary artery calcium measurement and assessment

We collected non-ECG-gated CAC data of patients at baseline and at 3, 6, and 12 months (± 30 days) after treatment from chest CT scans (Additional file 1: Fig. S1). Imaging parameters were as follows: slice thickness, 1.5 to 2.0 mm; tube current, modulated automatically; tube voltage, 120 kV; and image reconstruction, standard soft convolution kernel. Two independent radiologists (Zheng C. S. and Yang M. with 26 and 8 years of cardiovascular imaging experience, respectively) manually outlined the region of interest in four coronary arteries (the left main trunk, left anterior descending artery, circumflex, and right coronary artery) utilizing semiautomatic software (CaScoring, Syngo, Siemens Healthineers). The software automatically calculated the CAC volume and score in each coronary artery and the total (with an attenuation ≥ 130 HU and an area ≥ 1.0 mm^2^). CAC score adopts those described by Agatston et al. [26]. Changes in CAC volume and scores were measured to absolute progression (3, 6, 12 months data—baseline data) and relative progression $\left(\frac{3,\;6,\;12\;\mathrm{months}\;\mathrm{data}\;-\;\mathrm{baseline}\;\mathrm{data}}{3,\;6,\;12\;\mathrm{months}}\right)$. In addition, according to the Agatston score, the CAC results were stratified as mild calcification (CAC score = 1–99), moderate calcification (CAC score = 100–400), and severe calcification (CAC score > 400) [24]. A third radiologist subjectively classified the image quality of the patient as good, moderate, or poor due to motion artifacts and excluded patients with poor image quality at any time. Furthermore, we randomly selected patients with two CT scans less than 30 days apart, and there was the excellent interscan agreement for CAC score (intraclass correlation [ICC] = 0.92; 95% confidence interval [CI], 0.86–0.96) and volume (*ICC* = 0.95; 95% *CI*, 0.91–0.97). All three readers were blinded to patients’ clinical information. Any disagreements were resolved through group discussion.

### Follow-up and endpoints

Patients underwent repeated imaging examinations during outpatient or inpatient follow-up visits before ICI or non-ICI treatment and 3, 6, and 12 months after treatment. The primary endpoint of this study was the occurrence of ASCVD events, which included myocardial infarction, coronary revascularization, and ischemic stroke. Potential events were assessed through the review of imaging, medical records for hospitalizations and outpatients, death certificates, and discharge summaries. A telephonic interview was conducted with each patient or a family member. Four experienced radiologists and cardiologists independently adjudicated each event based on standardized definitions described by Drobni et al. [14] and the 2013 ACC/AHA Guidelines on the Assessment of Cardiovascular Risk [27]. They were blinded to all other data. Any disagreements were reconfirmed through interviews with the next of kin and finalized through discussion and negotiation. Secondary endpoints included the progression of CAC volume and score after treatment. All patients were followed up until April 2023 or death.

### Statistical analysis

The normal distribution of variables was assessed by the Kolmogorov–Smirnov test. Continuous variables were presented as mean (SD) or median (IQR) and categorical variables as counts (percentages). Continuous variables were compared using the *t*-test or Wilcoxon rank-sum test, whereas categorical variables were compared using the Pearson *χ*^2^ test or Fisher’s exact test. Cumulative incidence curves were constructed using the Kaplan–Meier method, and differences were evaluated by the log-rank test. To determine risk factors for ASCVD events, we performed univariable and multivariable regression analyses using Cox proportional hazards model. Except for cases with special instructions, all parameters with *P* < 0.10 in the univariable analysis were included in the multivariable model, and hazard ratios (HR) and 95% CIs were calculated. We also conducted exploratory subgroup analyses based on covariates of clinical interest. HRs with 95% CIs were reported within each subgroup. Imaging data were analyzed at baseline and 3, 6, and 12 months after treatment. The progression of the CAC volume and score between the ICI and non-ICI groups was compared using the Wilcoxon rank-sum test, and the progression of the CAC score grade between the two groups was compared using the chi-square test. Three multiple linear regression models were used to evaluate the association between ICIs and CAC and reported as the correlation coefficient (beta) adjusted for different covariates. Spearman rank correlation was used for correlation scatter plots of cycles of ICI versus the progression of the CAC volume and score. Propensity score matching (PSM) was performed in a 1:1 ratio based on all baseline characteristics to reduce potential confounding factors with the caliper value of 0.05. A *P*-value < 0.05 was considered statistically significant. Statistical analyses were performed using SPSS (version 26.0; IBM Corp) and R (version 4.3.0; R Foundation) statistical software.

## Results

### Patient characteristics

The ASCVD event study involved a total of 1458 patients, including 487 (33.4%) patients who received ICI therapy and 971 (66.6%) patients who did not. After PSM, there were 446 patients in each group. The baseline demographic characteristics of the patients are shown in Table 1, and the baseline laboratory values are presented in Additional file 1: Table S1. Overall, the proportion of male participants in the ICI group was higher than that in the non-ICI group (87.5% vs. 59.7%, *P* < 0.001). The ICI group had higher proportions of patients aged < 65 years (46.0% vs. 38.6%, *P* = 0.007) and patients with smoking index > 400 (41.7% vs. 19.8%, *P* < 0.001). In the ICI group, the proportions of adenocarcinoma and squamous cell carcinoma were comparable, whereas, in the non-ICI group, the most dominant pathological type was adenocarcinoma (44.6% and 48.3% vs. 76.4% and 18.0%, respectively; *P* < 0.001). Patients in the non-ICI group had more advanced tumor clinical stages (stage IV, 81.9% vs. 71.7%, *P* < 0.001). After PSM, there were no statistically significant differences in the baseline factors among the two groups. In addition, in the ICI group, PD-1 antibodies were the most commonly used antibodies, followed by PD-L1 antibody. All treatments exceeded four cycles.

**Table 1:** Baseline characteristics of patients in ASCVD events study before and after PSM

Parameter	Before PSM			After PSM		
	ICI group	Non-ICI group	*p*-value	ICI group	Non-ICI group	*p*-value
Patients, *n*	487	971		446	446	
Sex, *n* (%)			< 0.001			0.693
Male	426 (87.5%)	580 (59.7%)		385 (86.3%)	389 (87.2%)	
Female	61 (12.5%)	391 (40.3%)		61 (13.7%)	57 (12.8%)	
Age, *n* (%)			0.007			0.460
< 65	224 (46.0%)	375 (38.6%)		243 (54.5%)	232 (52.0%)	
≥ 65	263 (54.0%)	596 (61.4%)		203 (45.5%)	214 (48.0%)	
Body mass index (kg/m^2^), mean (SD)	22.4 (3.0)	22.6 (3.0)	0.115	22.4 (3.0)	22.6 (3.0)	0.528
Cancer characteristics, *n* (%)						
Stages			< 0.001			0.821
Stage III	138 (28.3%)	176 (18.1%)		121 (27.1%)	118 (26.5%)	
Stage IV	349 (71.7%)	795 (81.9%)		325 (72.9%)	328 (73.5%)	
Pathological types			< 0.001			0.108
Adenocarcinoma	217 (44.6%)	742 (76.4%)		216 (48.4%)	247 (55.4%)	
Squamous cell carcinoma	235 (48.3%)	175 (18.0%)		196 (43.9%)	167 (37.4%)	
Other^a^	35 (7.2%)	54 (5.6%)		34 (7.6%)	32 (7.2%)	
Cardiovascular risk factors, *n* (%)						
Hypertension	152 (31.2%)	313 (32.2%)	0.693	144 (32.3%)	171 (38.3%)	0.059
Diabetes	45 (9.2%)	63 (6.5%)	0.058	39 (8.7%)	42 (9.4%)	0.727
Smoking index, *n* (%)			< 0.001			0.270
≤ 400	284 (58.3%)	779 (80.2%)		268 (60.1%)	284 (63.7%)	
> 400	203 (41.7%)	192 (19.8%)		178 (39.9%)	162 (36.3%)	
Hyperlipidemia	68 (14.0%)	169 (17.4%)	0.093	65 (14.6%)	59 (13.2%)	0.561
History of cardiovascular disease, *n* (%)	47 (9.7%)	68 (7.0%)	0.077	42 (9.4%)	49 (11.0%)	0.439
Cardiovascular medications, *n* (%)						
Statins	35 (7.2%)	59 (6.1%)	0.415	31 (7.0%)	36 (8.1%)	0.525
Aspirin	24 (4.9%)	47 (4.8%)	0.941	22 (4.9%)	21 (4.7%)	0.876
Other antiplatelet therapies	19 (3.9%)	34 (3.5%)	0.700	17 (3.8%)	16 (3.6%)	0.859
Chest radiation therapy, *n* (%)	107 (22.0%)	174 (17.9%)	0.064	98 (22.0%)	93 (20.9%)	0.683
Cycles of ICI, mean (SD)	9.1 (7.7)			9.4 (7.9)		
ICIs type, *n* (%)						
PD-1 antibody	450 (92.4%)			411 (92.2%)		
PD-L1 antibody	35 (7.2%)			33 (7.4%)		
CTLA-4 antibody	2 (0.4%)			2 (0.4%)		

*Abbreviations*: *ASCVD* atherosclerotic cardiovascular disease, *ICI* immune checkpoint inhibitor, *SD* standard deviation, *PD-1* programmed cell death protein 1, *PD-L1* programmed cell death ligand 1, *CTLA-4* cytotoxic T-lymphocyte-associated protein 4, *PSM* propensity score matching

^a^Non-small cell lung cancer other than squamous cell carcinoma and adenocarcinoma

### Cardiovascular endpoints

**Fig. 2:**
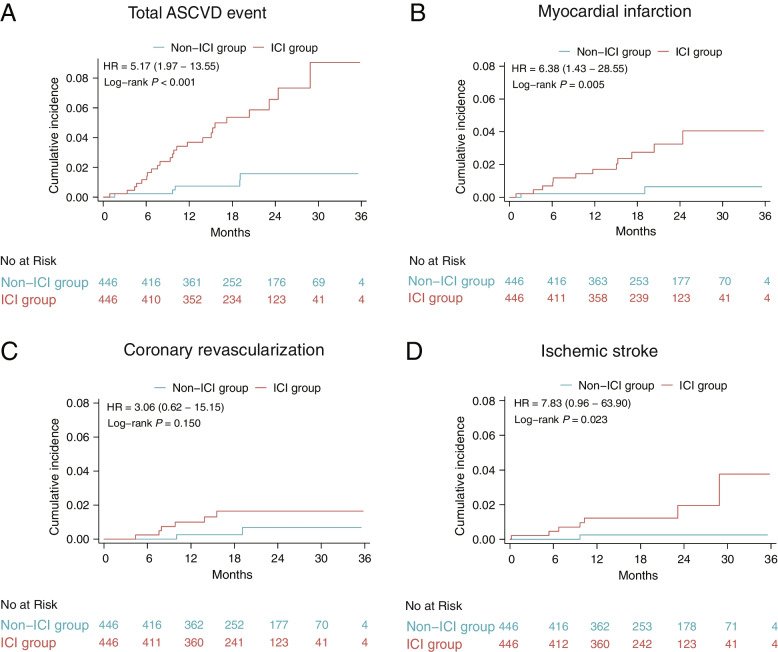
Kaplan–Meier curves of the cumulative incidence of the ASCVD events between the ICI and the non-ICI group after PSM. (**A**) Cumulative incidence for the total ASCVD event. (**B**-**D**) Cumulative incidence for the individual components of the ASCVD events. Abbreviations: HR, hazard ratio; ASCVD, atherosclerotic cardiovascular disease; ICI, immune checkpoint inhibitor; PSM, propensity score matching

A total of 24 ASCVD events (4.9%) occurred in the ICI group during entire period of follow-up (median follow-up 23.1 months), including 12 myocardial infarctions (2.5%), 6 coronary revascularizations (1.2%), and 7 ischemic strokes (1.4%). A total of 14 ASCVD events (1.4%) occurred in the non-ICI group, including 6 myocardial infarctions (0.6%), 4 coronary revascularizations (0.4%), and 4 ischemic strokes (0.4%). After PSM, 24 (5.4%) and 5 (1.1%) ASCVD events occurred in the ICI and non-ICI groups, respectively. The event rates for the total ASCVD and each endpoint increased after ICIs therapy, both before and after PSM. The cumulative incidence of the total and individual component endpoints in the ICI and non-ICI groups is depicted by Kaplan–Meier curves in Additional file 1: Fig. S2 and Fig. 2. In univariable analysis, smoking index, history of cardiovascular disease, neutrophil-to-lymphocyte ratio, and aspirin use were identified as potential predictors, where ICI therapy was associated with a 3.6-fold increase in the total risk of ASCVD events before PSM (*HR*, 3.6 [95% *CI*, 1.8–6.9]; *P* < 0.001). These covariates were further incorporated into the multivariable Cox regression model, showing that the correlation between ICI therapy and ASCVD events was attenuated but still significant (*HR*, 3.0 [95% *CI*, 1.5–6.0]; *P* = 0.002) (Additional file 1: Table S2). After PSM, ICIs, smoking index, and history of cardiovascular disease were identified as potential risk factors to include into multivariable analysis. Finally, ICIs (*HR*, 5.1 [95% *Cl*, 1.9–13.4]; *P* < 0.001) and history of cardiovascular disease (*HR*, 2.8 [95% *Cl*, 1.8–6.7]; *P* = 0.020) were independent risk factors associated with higher risk of ASCVD events (Table 2). We performed independent Cox regression analysis on lipids and cycles of ICI; however, there were no positive findings (Additional file 1: Table S3, Table S4, Table S5). Additionally, EGFR was identified as a potentially protective factor for ASCVD events in univariable analysis (*HR*, 0.3 [95% *Cl*, 0.1–0.9]; *P* = 0.035). We did not find an association between ALK and KRAS with ASCVD events (Additional file 1: Table S6). Increasing risk of ASCVD events with ICI therapy was consistently observed in the subgroups based on baseline characteristics both before and after PSM (Additional file 1: Fig. S3, Fig. S4). Compared with males, the risk of ASCVD events caused by ICIs was relatively higher in females before PSM (*HR*, 12.6 [95% *CI*, 3.0–53.1] vs. 2.4 [95% *CI*, 1.1–5.0]; *P* = 0.050) (Additional file 1: Fig. S3). However, after PSM, this difference did not reach statistical significance (*HR*, 5.3 [95% *CI*, 0.6–45.5] vs. 5.1 [95% *CI*, 1.7–14.9]; *P* = 0.979) (Additional file 1: Fig. S4). We compared the survival status of patients with and without ASCVD events and found that patients without ASCVD events had higher survival rates in half a year, 1 year, and 2 years, although this did not reach statistical significance (Additional file 1: Table S7).

**Table 2:** Univariate and multivariate Cox proportional hazards analyses of ASCVD events after PSM

Parameter	Univariate analysis		Multivariate analysis	
	Hazard ratio (95% *CI*)	*p*-value	Hazard ratio (95% *CI*)	*p*-value
ICIs				
No	Reference			
Yes	5.166 (1.969, 13.553)	< 0.001	5.104 (1.942, 13.416)	< 0.001
Sex				
Female	Reference			
Male	0.602 (0.245, 1.477)	0.268		
Age				
< 65	Reference			
≥ 65	1.775 (0.847, 3.718)	0.128		
Body mass index (kg/m^2^)	0.942 (0.830, 1.069)	0.353		
Hypertension				
No	Reference			
Yes	1.580 (0.759, 3.279)	0.221		
Diabetes				
No	Reference			
Yes	0.819 (0.195, 3.446)	0.786		
Smoking index				
≤ 400	Reference			
> 400	2.066 (0.993, 2.292)	0.052	1.709 (0.810, 3.610)	0.159
Hyperlipidemia				
No	Reference			
Yes	0.957 (0.333, 2.751)	0.936		
History of cardiovascular disease				
No	Reference			
Yes	2.981 (1.273, 6.983)	0.012	2.801 (1.176, 6.671)	0.020
Stages				
Stage III	Reference			
Stage IV	1.511 (0.632, 3.610)	0.354		
Chest radiation therapy				
No	Reference			
Yes	1.580 (0.719, 3.471)	0.254		
Statins				
No	Reference			
Yes	0.931 (0.221, 3.921)	0.923		
Aspirin				
No	Reference			
Yes	2.564 (0.776, 8.477)	0.123		
Hemoglobin (g/L)	1.000 (0.979, 1.022)	0.997		
Neutrophil-to-lymphocyte ratio	1.013 (0.961, 1.068)	0.632		
Platelet count-to-lymphocyte ratio	1.000 (0.997, 1.002)	0.763		

All hazard ratios are shown for one-unit increments for each variable except for categorical variables

*Abbreviations*: *ASCVD* atherosclerotic cardiovascular disease, *ICI* immune checkpoint inhibitor, *CI* confidence interval, *PSM* propensity score matching

### Progression of coronary artery calcium

A total of 246 patients (113 with ICI therapy and 133 without) were further enrolled in the CAC imaging study cohort. After PSM, 150 patients with balanced baseline characteristics were enrolled in the two groups at a 1:1 ratio. Their baseline characteristics (Additional file 1: Table S8, Table S9) were similar to those in the cardiovascular study cohort. All these patients had calcified coronary artery plaques before treatment, and baseline CAC volume and score did not differ between the two groups both before and after PSM.

**Fig. 3:**
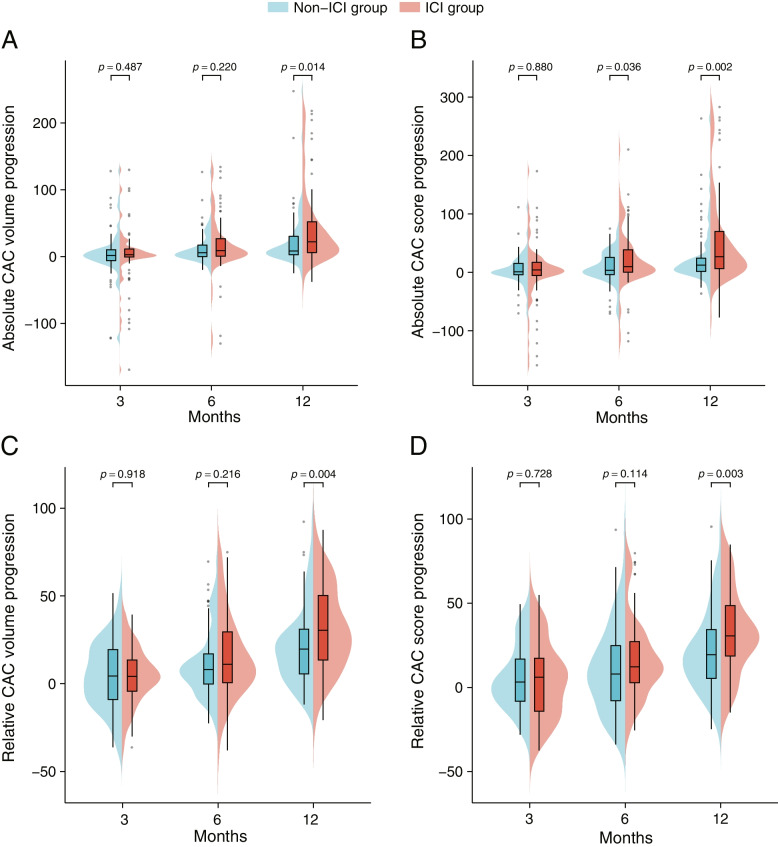
Bean plots show time-course progression of the CAC volume and score between the ICI and the non-ICI group after PSM. (**A**) Absolute CAC volume progression. (**B**) Absolute CAC score progression. (**C**) Relative CAC volume progression. (**D**) Relative CAC score progression. Abbreviations: CAC, coronary artery calcium; ICI, immune checkpoint inhibitor; PSM, propensity score matching

The patients underwent chest CT examinations 3, 6, and 12 months after treatment, and their CAC data at the four time points are summarized in Additional file 1: Table S10 and Table S11. Overall, we observed that the ICI group had significantly worse CAC volume and score progression than the non-ICI group did. Bean plots presents the variances in CAC progression in the two groups at the four time points before (Additional file 1: Fig. S5) and after PSM (Fig. 3). Specifically, there was no difference in CAC volume and score progression between the two groups at 3 months after treatment, but differences in volume and score progression emerged at 6 months, and absolute volume progression (29.2 mm^3^ vs. 10.1 mm^3^; *P* < 0.001 before PSM; 22.1 mm^3^ vs. 8.3 mm^3^; *P* = 0.013 after PSM), absolute score progression (34.3 vs. 12.8; *P* < 0.001 before PSM; 26.6 vs. 12.4; *P* = 0.002 after PSM), relative volume progression (27.4% vs. 20.1%; *P* = 0.014 before PSM; 30.4% vs. 19.7%; *P* = 0.004 after PSM), and relative score progression (30.6% vs. 21.2%; *P* = 0.016 before PSM; 30.6% vs. 19.4%; *P* = 0.003 after PSM) of CAC showed significant difference at 12 months after treatment (Additional file 1: Table S12, Table 3). Each coronary artery segment was analyzed independently. Their CAC progression trend was similar to that of the total population, with left anterior descending artery being the most prominent (Additional file 1: Fig. S6A). A similar trend appeared after PSM (Additional file 1: Fig. S6B). In addition, we quantified the CAC score at each time point as the grade representing calcification severity, according to the description of Budoff et al. [24] (Additional file 1: Table S13). The CAC score grade progression was significantly more severe in the ICI group than in the non-ICI group and showed statistically significant difference at 12 months (16.8% vs. 6.8%; *P* = 0.013 before PSM; 20.0% vs. 8.0%; *P* = 0.032 after PSM) (Additional file 1: Table S14, Fig. S7). In the multiple linear regression analysis, ICI therapy was a significant independent predictor of absolute volume progression, absolute score progression, relative volume progression, and relative score progression of CAC at 12 months across all models (absolute volume progression before PSM was marginally significant in model 2), both before and after PSM (Additional file 1: Table S15, Table 4). We performed Spearman correlation analysis on ICI cycles and CAC progression. However, an association between these two variables was not established (Additional file 1: Fig. S8).

**Table 3:** Progression of CAC volume and score between the ICI and the non-ICI group at 12 months after PSM

	ICI group (IQR)	Non-ICI group (IQR)	*p*-values*
Absolute volume progression (mm^3^)	22.1 (6.2, 52.1)	8.3 (2.8, 30.2)	0.013
Absolute score progression	26.6 (6.6, 69.9)	12.4 (1.6, 24.2)	0.002
Relative volume progression (%)	30.4 (13.5, 50.3)	19.7 (5.6, 31.0)	0.004
Relative score progression (%)	30.6 (18.7, 48.6)	19.4 (5.3, 34.3)	0.003

*Abbreviations*: *CAC* coronary artery calcium, *ICI* immune checkpoint inhibitor, *IQR* interquartile range, *PSM* propensity score matching

^*^Wilcoxon rank-sum test comparing progression of CAC volume and score between the ICI group with the non-ICI group

**Table 4:** Multiple linear regression analysis for associations of ICIs with absolute volume progression, absolute score progression, relative volume progression, and relative score progression of CAC at 12 months after PSM

	Model 1		Model 2		Model 3	
	Beta (SE)	*p*-value	Beta (SE)	*p*-value	Beta (SE)	*p*-value
Absolute volume progression	0.197 (0.081)	0.015	0.191 (0.082)	0.020	0.165 (0.061)	0.007
Absolute score progression	0.240 (0.081)	0.003	0.238 (0.083)	0.004	0.188 (0.061)	0.002
Relative volume progression	0.223 (0.081)	0.006	0.213 (0.083)	0.010	0.244 (0.079)	0.002
Relative score progression	0.224 (0.082)	0.006	0.232 (0.083)	0.005	0.233 (0.081)	0.004

*Abbreviations*: *ICI* immune checkpoint inhibitor, *CAC* coronary artery calcium, *SE* standard error, *PSM* propensity score matching

Model 1 is adjusted for sex, age, and history of cardiovascular disease

Model 2 is adjusted for body mass index, hypertension, diabetes, smoking index, hyperlipidemia, chest radiation therapy, statins, aspirin, hemoglobin, neutrophil-to-lymphocyte ratio, and platelet count to lymphocyte ratio in addition to the variables in the model 1

Model 3 is adjusted for baseline coronary artery calcium volume and score, in addition to the variables in the model 2

## Discussion

The relationship between immune checkpoint therapies and atherosclerosis is complex. The natural “brakes” in the immune system, including CTLA-4, PD-1, and PD-L1, plays an important role in maintaining the homeostasis of the immune system by limiting the magnitude of the immune response [28]. However, tumors utilize these checkpoints to protect themselves from immune attack [29]. Consequently, several checkpoint inhibitors have been developed to block this inhibitory pathway [30] and enhance the T-cell-mediated antitumor immune responses [31]. However, not only T cells playing anti-tumor roles in tumors or lymph nodes were activated but also the T cells in the immune system were activated. These “off-target” T cells may elicit preexisting autoimmune diseases or acute irAEs and even aggravate chronic inflammatory diseases [32]. Atherosclerosis is a long-term inflammatory condition triggered by the accumulation of immune-cell and lipid-rich plaques in the artery wall [33]. T cells were found to be the predominant cell type in both human and mouse atherosclerotic lesions by single-cell RNA sequencing and mass cytometry [34, 35]. The activation, differentiation, and exhaustion of T cells in plaques were more pronounced compared to their blood counterparts. These cells promote the development of vulnerable atherosclerotic plaques by perforin- and granzyme B-mediated apoptosis of macrophages, smooth muscle cells, and endothelial cells [36]. High quantities of PD-1 were expressed by the depletion of T cell, indicating that PD-1 antibodies could reactivate these exhausted cells, consequently driving the development of vulnerable plaques that contributed to the rupture of the plaques, formation of a thrombi, and acute vascular occlusion [37]. In addition to targeting the PD-1–PD-L1 to directly activate T cells, blocking CTLA4 could also induce an activated CD4 and CD8 T cells in the bloodstream and promote activation of the aortic endothelium [32].

In our study, we compared the rates of ASCVD events that occurred during the follow-up period in patients with NSCLC between two groups while assessing changes in CAC volume and score over time in a subset of patients based on chest CT scans. In contrast to the non-ICI group, the ICI group had a considerably greater incidence of ASCVD events. Differences were observed in the volume and score progression 6 months after treatment, and at 12 months, there were statistically significant differences in all observable markers between the groups. These results suggest that atherosclerosis and related cardiovascular events should not be ignored during ICIs treatment and are likely to be potentially unrecognized irAEs.

The application of ICIs in cancer therapy has been expanding since ipilimumab, the first ICI, was authorized for the treatment of melanoma in 2011 [38]. However, irAEs were also encountered post-treatment. Previously, cardiovascular events related irAEs included mainly myocarditis, pericarditis, vasculitis, and arrhythmias [39–42], particularly fulminant myocarditis, as reported by Johnson et al. [43] in 2016. In 2020, Drobni et al. [14] first reported that ICIs were highly associated with atherosclerotic cardiovascular events. Recently, Wang et al. [15] found an increased risk of ASCVD events in patients with high-risk or advanced melanoma after ICI therapy. Similar conclusions have been reached in a cohort of patients with NSCLC. A new cancer diagnosis is also independently linked to a considerably elevated risk of cardiovascular death (*HR*, 1.33) and nonfatal morbidity, including stroke (*HR*, 1.44), heart failure (*HR*, 1.62), and pulmonary embolism (*HR*, 3.43), according to researchers from the University of Alberta [44]. This implies that patients with cancer have a higher chance of developing cardiovascular disease. Therefore, clinicians should be mindful of the combined hazards of tumors and immunotherapy for cardiovascular events in patients.

To the best of our knowledge, this study is the first to document CAC progression over time in cancer patients treated with ICIs. Coronary atherosclerosis is a manifestation of systemic atherosclerosis and often presents as CAC [45]. The presence and severity of CAC are closely related to ASCVD and future cardiovascular risk [17–20]. The formation and expansion of CAC are two stages of the disease, and their pathophysiological mechanisms are different [46, 47]. Our study focused on the impact of ICIs on CAC progression. We found that the progression of CAC was higher in the ICI group, and the annual progression rate of CAC in the non-ICI group was consistent with that shown in previous studies [48–50]. The association between the utilization of ICIs and atherosclerotic plaques has been observed previously. The rate of progression of total aortic plaque volume was > threefold higher after the start of ICI therapy in an imaging sub-study of 40 melanoma patients [14]. However, the number of participants in imaging study was limited, and only two-time points (before ICIs and after ICIs) were selected for the analysis. The total plaque volume was adjusted at varying time intervals between scans to create an annualized rate of plaque volume change. The time point at which the CT scan was performed varied in each patient, but plaque progression was not linear. Therefore, the annualized rate of change may be biased, and short-term changes in plaque progression were not assessed. Based on this study, the number of patients was increased by more than fivefold to 246 participants in our imaging cohort study, and the progression of CAC was unadjusted, reflecting the real change at each time point. Researchers discovered that CV events following ICI therapy were linked to worse coronary and aortic calcifications on baseline CT imaging [16]. In addition, we found that CAC progression in the left anterior descending artery was the most significant among all the coronary arterial branches, which concurs with the findings of the study by Mohammad et al. [51] study in a population with coronary artery stenosis. Blood pressure, turbulent flow, or variations in the reactions of various coronary arterial branches to ICIs may all contribute to this outcome. However, in our study, we did not find an association between smoking and ASCVD events. We suspect this is because two-thirds (16/24) of ASCVD events occurred within 12 months after initiating ICIs, but the impact of smoking on cardiovascular events is a long-term, chronic process, and therefore, smoking-related cardiovascular events are not concentrated in 1 or 2 years [52, 53].

This study has a few limitations. First, as a retrospective cohort study, heterogeneity existed between the originally enrolled cases and controls. To address this issue, we used PSM to balance the baseline clinical and demographic characteristics between the two groups. Second, the CAC volume and score were obtained using non-ECG-gated chest CT and may have easily been affected by respiratory and heartbeat artifacts. To address this aspect, we manually classified the image quality as good, moderate, or poor and removed images with poor image quality, such as heavy artifacts, and the interscan agreement for CAC score (*ICC* = 0.92; 95% *CI*, 0.86–0.96) and volume (*ICC* = 0.95; 95% *CI*, 0.91–0.97) was excellent. In addition, we quantified the CAC score as a grade representing the severity of calcification, which was demonstrated to be reliable in non-gated CT by Budoff et al. [24], and its trend of progression was similar to the CAC volume and score in both the ICI and non-ICI groups. Third, our follow-up period was not long enough because ICIs have been primarily used in patients with advanced cancer who cannot undergo operative management. Recently, a phase III clinical trial, KEYNOTE-671 (NCT03425643), showed that neoadjuvant pembrolizumab plus chemotherapy followed by resection and adjuvant pembrolizumab demonstrated a 42% reduction in the risk of cancer recurrence, progression, or mortality in patients with resectable, early-stage NSCLC [54]. This result indicates that ICI therapy will be used more frequently, and that patients with early- and mid-stage malignancies will have longer survival. It remains unclear, however, whether the adverse effects of ICIs on atherosclerosis will be amplified or diminished over time. Fourth, since most patients did not undergo coronary artery CTA, we only analyzed calcified plaques. Drobni et al. [55] recently found that ICIs are associated with aortic noncalcified plaque progression. However, we do not yet know whether plaques in the aorta (whether calcified or non-calcified) have similar clinical significance to plaques in the coronary arteries. Therefore, future studies are needed to further analyze the relationship between coronary artery non-calcified plaques and ICI use. Lastly, this was a retrospective, single-center, single-cancer study. There may be differences in the adverse reactions of different cancer types to ICIs, and the data of other irAEs was missing. Our study had the largest single-cancer cohort reported to date; however, more prospective multicenter studies with larger sample sizes are necessary.

## Conclusions

Our study revealed that patients receiving ICI therapy had a higher incidence of ASCVD events, and that the progress of CAC was considerably greater than what has been reported in the literature [48–50]. ICIs may increase cardiovascular events by accelerating the progression of systemic atherosclerosis, including that in the coronary arteries. Awareness of atherosclerotic adverse events occurring during immunotherapy should be improved, comprehensive screening for cardiovascular risk factors should be performed before initiating ICIs, and serial quantification of CAC on non-gated CT scans might be considered as a standard of practice.

### Supplementary Information

**Additional file 1: Table S1.** Baseline laboratory values of patients in ASCVD events study before and after PSM. **Table S2.** Univariate and Multivariate Cox proportional hazards analyses of ASCVD events before PSM. **Table S3.** Univariate and Multivariate Cox proportional hazards analyses of ASCVD events with blood lipids before PSM. **Table S4.** Univariate and Multivariate Cox proportional hazards analyses of ASCVD events with blood lipids after PSM. **Table S5.** Univariate and Multivariate Cox proportional hazards analyses of ASCVD events with the cycles of ICI. **Table S6.** Univariate and Multivariate Cox proportional hazards analyses of ASCVD events with EGFR, ALK and KRAS. **Table S7.** The survival rate of patients with and without ASCVD events before and after PSM. **Table S8.** Baseline characteristics of patients in imaging study before and after PSM. **Table S9.** Baseline laboratory values of patients in imaging study before and after PSM. **Table S10.** CAC volume and score in the ICI and the Non-ICI group at four time points before PSM. **Table S11.** CAC volume and score in the ICI and the Non-ICI group at four time points after PSM. **Table S12.** Progression of CAC volume and score Between the ICI and the Non-ICI Group at 12 Months before PSM. **Table S13.** Number of patients with different CAC score grade in the ICI and the Non-ICI group before and after PSM. **Table S14.** CAC score grade progression in the ICI and the Non-ICI group before and after PSM. **Table S15.** Multiple linear regression analysis for associations of ICIs with absolute volume progression, absolute score progression, relative volume progression and relative score progression of CAC at 12 months before PSM. **Fig. S1.** Representative axial CT images show the progression of CAC volume and score in patients with and without ICI therapy. **Fig. S2.** Kaplan–Meier curves of the cumulative incidence of the ASCVD events between the ICI and the non-ICI group before PSM. **Fig. S3.** Forest plot of the hazard ratios for the total ASCVD event stratified by different subgroups before PSM. **Fig. S4.** Forest plot of the hazard ratios for the total ASCVD event stratified by different subgroups after PSM. **Fig. S5.** Bean plots show time-course progression of the CAC volume and score between the ICI and the non-ICI group before PSM. **Fig. S6.** Heatmap of CAC progression in each segment of the coronary artery before (A) and after (B) PSM. **Fig. S7.** Stacked bar graphs of CAC grade progression in ICI (A) and non-ICI (B) group before PSM and in ICI (C) and non-ICI (D) group after PSM. **Fig. S8.** Scatter plots of cycles of ICI versus absolute volume progression, absolute score progression, relative volume progression and relative score progression of CAC at 12 months before (A-D) and after (E–H) PSM.

## Data Availability

All data required in the paper, including the primary raw data, are provided in the original manuscript and supplementary material file.

## Abbreviations

ASCVD: Atherosclerotic cardiovascular disease
AST: Aspartate transaminase
CAC: Coronary artery calcium
CI: Confidence interval
CTLA-4: Cytotoxic T-lymphocyte-associated protein 4
HR: Hazard ratio
ICI: Immune checkpoint inhibitor
IQR: Interquartile range
irAEs: Immune-related adverse events
NSCLC: Non-small cell lung cancer
PD-1: Programmed cell death 1
PD-L1: Programmed cell death-ligand 1
PSM: Propensity score matching
SD: Standard deviation
SE: Standard error
