# Supplementary material for: Immune checkpoint inhibitors in cancer: the increased risk of atherosclerotic cardiovascular disease events and progression of coronary artery calcium
Source: BMC Med. 2024 Jan 31;22:44. doi: 10.1186/s12916-024-03261-x (PMC10829401; doi:10.1186/s12916-024-03261-x)
Supplement: Supplementary file 1 — Additional file 1: Table S1. Baseline laboratory values of patients in ASCVD events study before and after PSM. Table S2. Univariate and Multivariate Cox proportional hazards analyses of ASCVD events before PSM. Table S3. Univariate and Multivariate Cox proportional hazards analyses of ASCVD events with blood lipids before PSM. Table S4. Univariate and Multivariate Cox proportional hazards analyses of ASCVD events with blood lipids after PSM. Table S5. Univariate and Multivariate Cox proportional hazards analyses of ASCVD events with the cycles of ICI. Table S6. Univariate and Multivariate Cox proportional hazards analyses of ASCVD events with EGFR, ALK and KRAS. Table S7. The survival rate of patients with and without ASCVD events before and after PSM. Table S8. Baseline characteristics of patients in imaging study before and after PSM. Table S9. Baseline laboratory values of patients in imaging study before and after PSM. Table S10. CAC volume and score in the ICI and the Non-ICI group at four time points before PSM. Table S11. CAC volume and score in the ICI and the Non-ICI group at four time points after PSM. Table S12. Progression of CAC volume and score Between the ICI and the Non-ICI Group at 12 Months before PSM. Table S13. Number of patients with different CAC score grade in the ICI and the Non-ICI group before and after PSM. Table S14. CAC score grade progression in the ICI and the Non-ICI group before and after PSM. Table S15. Multiple linear regression analysis for associations of ICIs with absolute volume progression, absolute score progression, relative volume progression and relative score progression of CAC at 12 months before PSM. Fig. S1. Representative axial CT images show the progression of CAC volume and score in patients with and without ICI therapy. Fig. S2. Kaplan–Meier curves of the cumulative incidence of the ASCVD events between the ICI and the non-ICI group before PSM. Fig. S3. Forest plot of the hazard ratios for the total ASCVD event st [file 12916_2024_3261_MOESM1_ESM.docx]

**SUPPLEMENTAL MATERIAL**

**Immune checkpoint inhibitors in cancer: the increased risk of** **atherosclerotic cardiovascular disease events and progression of** **coronary artery calcium**

Bingxin Gong,^1,2^ Yusheng Guo, ^1,2^ Yi Li,^1,2^ Jing Wang,^1,2^ Guofeng Zhou,^1,2^ Yong-hao Chen,^3^ Tong Nie, ^1,2^ Ming Yang,^1,2^ Kun Luo,^1,2^ Chuansheng Zheng,^1,2^ Feng Pan,^1,2^ Bo Liang,^1,2^ Lian Yang^1,2^

^1^Department of Radiology, Union Hospital, Tongji Medical College, Huazhong University of Science and Technology, Wuhan 430022, China.

^2^Hubei Key Laboratory of Molecular Imaging, Wuhan 430022, China.

^3^Department of Cardiology, State Key Laboratory of Complex Severe and Rare Diseases, Peking Union Medical College Hospital, Chinese Academy of Medical Sciences and Peking Union Medical College, Beijing 100005, China.

BXG, YSG and YL contributed equally.

Corresponding authors: Lian Yang, Department of Radiology, Union Hospital, Tongji Medical College, Huazhong University of Science and Technology, No.1277 Jiefang Avenue, Wuhan 430022, China; E-mail: yanglian@hust.edu.cn; Bo Liang, Department of Radiology, Union Hospital, Tongji Medical College, Huazhong University of Science and Technology, No.1277 Jiefang Avenue, Wuhan 430022, China; E-mail: xiehelb@sina.com.

**Table of Contents: page**

**Supplemental Tables**

• Table S1 Baseline laboratory values of patients in ASCVD events study before and after PSM……………………….………4

• Table S2 Univariate and Multivariate Cox proportional hazards analyses of ASCVD events before PSM………….…………5

• Table S3 Univariate and Multivariate Cox proportional hazards analyses of ASCVD events with blood lipids before PSM….6

• Table S4 Univariate and Multivariate Cox proportional hazards analyses of ASCVD events with blood lipids after PSM……7

• Table S5 Univariate and Multivariate Cox proportional hazards analyses of ASCVD events with the cycles of ICI…………..8

• Table S6 Univariate and Multivariate Cox proportional hazards analyses of ASCVD events with EGFR, ALK and KRAS…..9

• Table S7 The survival rate of patients with and without ASCVD events before and after PSM…………………………….…10

• Table S8 Baseline characteristics of patients in imaging study before and after PSM……………………………………….…11

• Table S9 Baseline laboratory values of patients in imaging study before and after PSM…………………………….………...12

• Table S10 CAC volume and score in the ICI and the Non-ICI group at four time points before PSM…………………………13

• Table S11 CAC volume and score in the ICI and the Non-ICI group at four time points after PSM…………………………...14

• Table S12 Progression of CAC volume and score Between the ICI and the Non-ICI Group at 12 Months before PSM………15

• Table S13 Number of patients with different CAC score grade in the ICI and the Non-ICI group before and after PSM….….16

• Table S14 CAC score grade progression in the ICI and the Non-ICI group before and after PSM…………………………….17

• Table S15 Multiple linear regression analysis for associations of ICIs with absolute volume progression, absolute score progression, relative volume progression and relative score progression of CAC at 12 months before PSM…………………….18

**Supplemental Figures and Figure Legends**

• Fig. S1 Representative axial CT images show the progression of CAC volume and score in patients with and without ICI therapy. Quantitative CT of coronary calcified plaque (red) at baseline and at 3 months, 6 months, and 12 months after treatment.

**(A)** an adult patient treatment with PD-1 antibody, and **(B)** an adult patient without ICI therapy. Abbreviations: CAC, coronary

artery calcium; ICI, immune checkpoint inhibitor; PD-1, programmed cell death ligand 1.……………………………………...19

• Fig. S2 Kaplan–Meier curves of the cumulative incidence of the ASCVD events between the ICI and the non-ICI group before PSM. **(A)** Cumulative incidence for the total ASCVD event. **(B-D)** Cumulative incidence for the individual components of the ASCVD events. Abbreviations: HR, hazard ratio; ASCVD, atherosclerotic cardiovascular disease; ICI, immune checkpoint inhibitor; PSM, propensity score matching.………………………………………………………………………………….……20

• Fig. S3 Forest plot of the hazard ratios for the total ASCVD event stratified by different subgroups before PSM. Hazard

ratios were derived from univariate Cox model for each subgroup. Dashed line indicates HR of 1. Abbreviations: ASCVD, atherosclerotic cardiovascular disease; PSM, propensity score matching.………………………………………………………..21

• Fig. S4 Forest plot of the hazard ratios for the total ASCVD event stratified by different subgroups after PSM. Hazard

ratios were derived from univariate Cox model for each subgroup. Dashed line indicates HR of 1. Abbreviations: ASCVD, atherosclerotic cardiovascular disease; PSM, propensity score matching.………………………………………………………..22

• Fig. S5 Bean plots show time-course progression of the CAC volume and score between the ICI and the non-ICI group before PSM. **(A)** Absolute CAC volume progression. **(B)** Absolute CAC score progression. **(C)** Relative CAC volume progression. **(D)** Relative CAC score progression. Abbreviations: CAC, coronary artery calcium; ICI, immune checkpoint inhibitor; PSM, propensity score matching.………………………………………………….........................................................................……..23

• Fig. S6 Heatmap of CAC progression in each segment of the coronary artery before **(A)** and after **(B)** PSM. Abbreviations: CAC, coronary artery calcium; PSM, propensity score matching; AVP, absolute volume progression; ASP, absolute score progression; RVP, relative volume progression; RSP, relative score progression; LM, left main trunk; LAD, left anterior descending artery; CX, circumflex; and RCA, right coronary artery……………………………………………………………..24

• Fig. S7 Stacked bar graphs of CAC grade progression in ICI **(A)** and non-ICI **(B)** group before PSM and in ICI **(C)** and non-ICI **(D)** group after PSM. Stacked bars of three different colors represent the proportion of different degrees of calcification in each group. Abbreviations: CAC, coronary artery calcium; ICI, immune checkpoint inhibitor; PSM, propensity score matching……25

• Fig. S8 Scatter plots of cycles of ICI versus absolute volume progression, absolute score progression, relative volume progression and relative score progression of CAC at 12 months before **(A-D)** and after **(E-H)** PSM. Abbreviations: ICI, immune checkpoint inhibitor; CAC, coronary artery calcium; PSM, propensity score matching………………………………………….26

**Table S1. Baseline laboratory values** **of patients in ASCVD events study before and after PSM**

| Laboratory parameters, mean (SD) |  | Before PSM |  |  |  | After PSM |  |
| --- | --- | --- | --- | --- | --- | --- | --- |
|  | ICI group | Non-ICI group | *P* value |  | ICI group | Non-ICI group | *P* value |
| Blood urea nitrogen (mmol/L) | 5.4 (1.7) | 5.3 (1.6) | 0.222 |  | 5.3 (1.7) | 5.5 (1.7) | 0.364 |
| Serum creatinine (μmol/L) | 72.4 (14.8) | 68.8 (17.3) | <0.001 |  | 72.3 (14.8) | 73.9 (19.0) | 0.177 |
| Total cholesterol (mmol/L) | 4.3 (1.0) | 4.4 (1.0) | 0.020 |  | 4.3 (1.0) | 4.3 (1.0) | 0.639 |
| Triglycerides (mmol/L) | 1.3 (0.7) | 1.5 (0.8) | <0.001 |  | 1.3 (0.7) | 1.3 (0.8) | 0.595 |
| High-density lipoprotein (mmol/L) | 1.1 (0.3) | 1.2 (0.3) | <0.001 |  | 1.1 (0.3) | 1.1 (0.3) | 0.921 |
| Low-density lipoprotein (mmol/L) | 2.6 (0.8) | 2.6 (0.7) | 0.745 |  | 2.6 (0.8) | 2.6 (0.8) | 0.647 |
| Hemoglobin (g/L) | 123.8 (16.3) | 124.2 (17.1) | 0.711 |  | 124.4 (16.2) | 125.0 (18.3) | 0.569 |
| Neutrophil to lymphocyte ratio | 5.6 (6.6) | 4.9 (5.3) | 0.054 |  | 5.7 (6.8) | 5.0 (5.2) | 0.093 |
| Platelet count to lymphocyte ratio | 214.6 (144.9) | 208.1 (140.6) | 0.410 |  | 215.5 (147.6) | 211.6 (143.8) | 0.691 |

Abbreviations: ASCVD, atherosclerotic cardiovascular disease; ICI, immune checkpoint inhibitor; SD, standard deviation; PSM, propensity score matching.

**Table S2. Univariate and Multivariate Cox proportional hazards analyses of ASCVD events before PSM**

| Parameter | Univariate analysis | |  | Multivariate analysis | |
| --- | --- | --- | --- | --- | --- |
|  | Hazard ratio (95% CI) | *P* value |  | Hazard ratio (95% CI) | *P* value |
| ICIs |  |  |  |  |  |
| No | Reference |  |  |  |  |
| Yes | 3.555 (1.827, 6.918) | <0.001 |  | 3.012 (1.511, 5.988) | 0.002 |
| Sex |  |  |  |  |  |
| Female | Reference |  |  |  |  |
| Male | 1.734 (0.793, 3.794) | 0.168 |  |  |  |
| Age |  |  |  |  |  |
| <65 | Reference |  |  |  |  |
| ≥65 | 1.149 (0.784, 2.849) | 0.222 |  |  |  |
| Body mass index (kg/m^2^) | 0.964 (0.865, 1.074) | 0.503 |  |  |  |
| Hypertension |  |  |  |  |  |
| No | Reference |  |  |  |  |
| Yes | 1.713 (0.894, 3.283) | 0.105 |  |  |  |
| Diabetes |  |  |  |  |  |
| No | Reference |  |  |  |  |
| Yes | 1.647 (0.583, 4.648) | 0.346 |  |  |  |
| Smoking index |  |  |  |  |  |
| ≤400 | Reference |  |  |  |  |
| >400 | 2.160 (1.127, 4.149) | 0.020 |  | 1.422 (0.720, 2.809) | 0.311 |
| Hyperlipidemia |  |  |  |  |  |
| No | Reference |  |  |  |  |
| Yes | 0.946 (0.394, 2.267) | 0.900 |  |  |  |
| History of cardiovascular disease |  |  |  |  |  |
| No | Reference |  |  |  |  |
| Yes | 3.451 (1.577, 7.551) | 0.002 |  | 2.341 (0.935, 5.858) | 0.069 |
| Stages |  |  |  |  |  |
| Stage III | Reference |  |  |  |  |
| Stage IV | 1.511 (0.615, 3.717) | 0.368 |  |  |  |
| Chest radiation therapy |  |  |  |  |  |
| No | Reference |  |  |  |  |
| Yes | 1.482 (0.717 - 3.061) | 0.288 |  |  |  |
| Statins |  |  |  |  |  |
| No | Reference |  |  |  |  |
| Yes | 0.886 (0.213, 3.683) | 0.867 |  |  |  |
| Aspirin |  |  |  |  |  |
| No | Reference |  |  |  |  |
| Yes | 3.372 (1.314, 8.657) | 0.011 |  | 1.973 (0.665, 5.848) | 0.220 |
| Hemoglobin (g/L) | 1.007 (0.988, 1.027) | 0.468 |  |  |  |
| Neutrophil to lymphocyte ratio | 1.038 (1.000, 1.078) | 0.050 |  | 1.035 (0.996, 1.076) | 0.078 |
| Platelet count to lymphocyte ratio | 1.001 (1.000, 1.003) | 0.134 |  |  |  |

Abbreviations: ASCVD, atherosclerotic cardiovascular disease; ICI, immune checkpoint inhibitor; CI, confidence interval; PSM, propensity score matching.

All hazard ratios are shown for one-unit increments for each variable except for categorical variables.

**Table S3. Univariate and Multivariate Cox proportional hazards analyses of ASCVD events with blood lipids before PSM**

| Parameter | Univariate analysis | |  | Multivariate analysis* | |
| --- | --- | --- | --- | --- | --- |
|  | Hazard ratio (95% CI) | *P* value |  | Hazard ratio (95% CI) | *P* value |
| Total cholesterol (mmol/L) | 0.906 (0.655, 1.255) | 0.554 |  | 1.377 (0.516, 3.674) | 0.523 |
| Triglycerides (mmol/L) | 0.775 (0.475, 1.264) | 0.307 |  | 0.776 (0.443, 1.359) | 0.375 |
| High-density lipoprotein (mmol/L) | 0.488 (0.162, 1.473) | 0.203 |  | 0.387 (0.083, 1.811) | 0.228 |
| Low-density lipoprotein (mmol/L) | 0.952 (0.617, 1.470) | 0.825 |  | 0.845 (0.279, 2.564) | 0.767 |

Abbreviations: ASCVD, atherosclerotic cardiovascular disease; CI, confidence interval; PSM, propensity score matching.

*Multivariate analysis is adjusted for sex, age and history of cardiovascular disease.

**Table S4. Univariate and Multivariate Cox proportional hazards analyses of ASCVD events with blood lipids after PSM**

| Parameter | Univariate analysis | |  | Multivariate analysis* | |
| --- | --- | --- | --- | --- | --- |
|  | Hazard ratio (95% CI) | *P* value |  | Hazard ratio (95% CI) | *P* value |
| Total cholesterol (mmol/L) | 1.018 (0.706, 1.470) | 0.922 |  | 1.251 (0.387, 4.049) | 0.708 |
| Triglycerides (mmol/L) | 0.923 (0.548, 1.556) | 0.764 |  | 0.936 (0.513, 1.707) | 0.828 |
| High-density lipoprotein (mmol/L) | 1.547 (0.443, 5.407) | 0.494 |  | 1.055 (0.182, 6.128) | 0.952 |
| Low-density lipoprotein (mmol/L) | 0.962 (0.594, 1.557) | 0.874 |  | 0.835 (0.221, 3.159) | 0.791 |

Abbreviations: ASCVD, atherosclerotic cardiovascular disease; CI, confidence interval; PSM, propensity score matching.

*Multivariate analysis is adjusted for sex, age and history of cardiovascular disease.

**Table S5. Univariate and Multivariate Cox proportional hazards analyses of ASCVD events with the cycles of ICI**

| Parameter | Univariate analysis | |  | Multivariate analysis* | |
| --- | --- | --- | --- | --- | --- |
|  | Hazard ratio (95% CI) | *P* value |  | Hazard ratio (95% CI) | *P* value |
| Cycles of ICI | 0.947 (0.883, 1.016) | 0.129 |  | 0.950 (0.885, 1.020) | 0.155 |

Abbreviations: ASCVD, atherosclerotic cardiovascular disease; ICI, immune checkpoint inhibitor; CI, confidence interval.

*Multivariate analysis is adjusted for sex, age and history of cardiovascular disease.

**Table S6. Univariate and Multivariate Cox proportional hazards analyses of ASCVD events with EGFR, ALK and KRAS**

| Parameter | Total(N) | Univariate analysis | |  | Multivariate analysis* | |
| --- | --- | --- | --- | --- | --- | --- |
|  |  | Hazard ratio (95% CI) | *P* value |  | Hazard ratio (95% CI) | *P* value |
| EGFR | 858 |  |  |  |  |  |
| - | 471 | Reference |  |  | Reference |  |
| + | 387 | 0.342 (0.126, 0.927) | 0.035 |  | 0.573 (0.192, 1.711) | 0.319 |
| ALK | 858 |  |  |  |  |  |
| - | 792 | Reference |  |  |  |  |
| + | 66 | 0.525 (0.071, 3.902) | 0.529 |  |  |  |
| KRAS | 858 |  |  |  |  |  |
| - | 785 | Reference |  |  |  |  |
| + | 73 | 1.879 (0.556, 6.352) | 0.310 |  |  |  |

Abbreviations: ASCVD, atherosclerotic cardiovascular disease; ICI, immune checkpoint inhibitor; CI, confidence interval.

*Multivariate analysis is adjusted for sex, age, history of cardiovascular disease and ICIs usage.

**Table S7. The survival rate of patients with and without ASCVD events before and after PSM**

| Time-points | Before PSM | | After PSM | |
| --- | --- | --- | --- | --- |
|  | Without ASCVD events | With ASCVD events | Without ASCVD events | With ASCVD events |
| Half-year survival rate | 94.7% | 89.4% | 93.8% | 89.7% |
| One-year survival rate | 86.2% | 76.3% | 84.1% | 79.3% |
| Two-year survival rate | 72.7% | 67.7% | 70.8% | 69.0% |

Abbreviations: ASCVD, atherosclerotic cardiovascular disease; PSM, propensity score matching.

**Table S8. Baseline characteristics of patients in imaging study before and after PSM**

| Parameter | Before PSM | | |  | After PSM | | |
| --- | --- | --- | --- | --- | --- | --- | --- |
|  | ICI group | Non-ICI group | *P* value |  | ICI group | Non-ICI group | *P* value |
| Patients, n | 113 | 133 |  |  | 75 | 75 |  |
| Sex, n (%) |  |  | < 0.001 |  |  |  | 0.497 |
| Male | 103 (91.2%) | 82 (61.7%) |  |  | 65 (86.7%) | 62 (82.7%) |  |
| Female | 10 (8.8%) | 51 (38.3%) |  |  | 10 (13.3%) | 13 (17.3%) |  |
| Age (y), n (%) |  |  | 0.348 |  |  |  | 0.870 |
| <65 | 62 (54.9%) | 65 (48.9%) |  |  | 37 (49.3%) | 36 (48%) |  |
| ≥65 | 51 (45.1%) | 68 (51.1%) |  |  | 38 (50.7%) | 39 (52%) |  |
| Body mass index (kg/m^2^), mean (SD) | 22.4 (3.2) | 23.1 (2.8) | 0.102 |  | 22.6 (3.6) | 22.9 (2.9) | 0.673 |
| Cancer characteristics, n (%) |  |  |  |  |  |  |  |
| Stage |  |  | 0.847 |  |  |  | 0.707 |
| Stage III | 26 (23.0%) | 32 (24.1%) |  |  | 18 (24.0%) | 20 (26.7%) |  |
| Stage IV | 87 (77.0%) | 101 (75.9%) |  |  | 57 (76.0%) | 55 (73.3%) |  |
| Pathological types |  |  | < 0.001 |  |  |  | 0.939 |
| Adenocarcinoma | 58 (51.3%) | 101 (75.9%) |  |  | 45 (60.0%) | 47 (62.7%) |  |
| Squamous cell carcinoma | 49 (43.4%) | 26 (19.5%) |  |  | 25 (33.3%) | 23 (30.7%) |  |
| Other* | 6 (5.3%) | 6 (4.5%) |  |  | 5 (6.7%) | 5 (6.7%) |  |
| Cardiovascular risk factors, n (%) |  |  |  |  |  |  |  |
| Hypertension | 61 (54.0%) | 77 (57.9%) | 0.538 |  | 41 (54.7%) | 41 (54.7%) | 1.000 |
| Diabetes | 17 (15.0%) | 16 (12.0%) | 0.489 |  | 10 (13.3%) | 9 (12%) | 0.806 |
| Smoking index, n (%) |  |  | 0.003 |  |  |  | 0.731 |
| ≤400 | 67 (59.3%) | 102 (76.7%) |  |  | 48 (64%) | 50 (66.7%) |  |
| >400 | 46 (40.7%) | 31 (23.3%) |  |  | 27 (36%) | 25 (33.3%) |  |
| Hyperlipidemia | 16 (14.2%) | 24 (18.0%) | 0.410 |  | 12 (16%) | 13 (17.3%) | 0.826 |
| History of cardiovascular disease, n (%) | 13 (11.5%) | 14 (10.5%) | 0.807 |  | 7 (9.3%) | 9 (12%) | 0.596 |
| Cardiovascular medications, n (%) |  |  |  |  |  |  |  |
| Statins | 15 (13.3%) | 16 (12.0%) | 0.769 |  | 5 (6.7%) | 5 (6.7%) | 1.000 |
| Aspirin | 8 (7.1%) | 9 (6.8%) | 0.923 |  | 1 (1.3%) | 2 (2.7%) | 0.900 |
| Other antiplatelet therapies | 5 (4.4%) | 3 (2.3%) | 0.552 |  | 1 (1.3%) | 3 (4%) | 0.612 |
| Chest radiation therapy, n (%) | 29 (25.7%) | 43 (32.3%) | 0.252 |  | 21 (28%) | 26 (34.7%) | 0.379 |
| Cycles of ICI, mean (SD) | 12.3 (8.0) |  |  |  | 12.7 (8.6) |  |  |
| ICIs type, n (%) |  |  |  |  |  |  |  |
| PD-1 antibody | 101 (89.4%) |  |  |  | 65 (86.7%) |  |  |
| PD-L1 antibody | 12 (10.6%) |  |  |  | 10 (13.3%) |  |  |
| CTLA-4 antibody | 0 (0.0%) |  |  |  | 0 (0.0%) |  |  |
| Radiographic analysis, median (IQR) |  |  |  |  |  |  |  |
| Coronary artery calcium volume (mm^3^) | 93.4 (37.8, 253.0) | 70.1 (27.4, 205.3) | 0.114 |  | 85.1 (28.2, 251.2) | 74.9 (27.45, 199.9) | 0.510 |
| Coronary artery calcium score | 96.9 (34.6, 306.8) | 68.8 (25.3, 226.6) | 0.103 |  | 76.4 (27.15, 286.5) | 76.8 (25.1, 224.15) | 0.480 |

Abbreviations: ICI, immune checkpoint inhibitor; SD, standard deviation; IQR, interquartile range; PD-1, programmed cell death protein 1; PD-L1, programmed cell death ligand 1; CTLA-4 cytotoxic T-lymphocyte-associated protein 4; PSM, propensity score matching.

*Non-small cell lung cancer other than squamous cell carcinoma and adenocarcinoma.

**Table S9. Baseline laboratory values** **of patients in imaging study before and after PSM**

| Laboratory parameters, mean (SD) |  | Before PSM |  |  |  | After PSM |  |
| --- | --- | --- | --- | --- | --- | --- | --- |
|  | ICI group | Non-ICI group | *P* value |  | ICI group | Non-ICI group | *P* value |
| Blood urea nitrogen (mmol/L) | 5.3 (1.6) | 5.5 (1.6) | 0.315 |  | 5.3 (1.6) | 5.4 (1.7) | 0.672 |
| Serum creatinine (μmol/L) | 73.8 (15.0) | 72.0 (16.4) | 0.383 |  | 74.9 (16.8) | 74.4 (15.8) | 0.721 |
| Total cholesterol (mmol/L) | 4.3 (1.0) | 4.5 (1.0) | 0.312 |  | 4.2 (1.0) | 4.3 (0.9) | 0.841 |
| Triglycerides (mmol/L) | 1.3 (0.6) | 1.5 (1.0) | 0.017 |  | 1.4 (0.7) | 1.4 (0.6) | 0.493 |
| High-density lipoprotein (mmol/L) | 1.1 (0.3) | 1.1 (0.3) | 0.080 |  | 1.1 (0.3) | 1.1 (0.3) | 0.802 |
| Low-density lipoprotein (mmol/L) | 2.7 (0.8) | 2.7 (0.7) | 0.990 |  | 2.5 (0.8) | 2.6 (0.8) | 0.785 |
| Hemoglobin (g/L) | 125.2 (15.4) | 122.6 (20.4) | 0.267 |  | 126.4 (16.8) | 125.6 (17.77) | 0.651 |
| Neutrophil to lymphocyte ratio | 4.9 (4.9) | 4.0 (3.6) | 0.093 |  | 4.8 (4.3) | 4.4 (4.8) | 0.773 |
| Platelet count to lymphocyte ratio | 211.2 (180.9) | 191.2 (106.0) | 0.283 |  | 211.9 (205.6) | 195.1 (96.0) | 0.608 |

Abbreviations: ICI, immune checkpoint inhibitor; SD, standard deviation; PSM, propensity score matching.

**Table S10. CAC volume and score in the ICI and the Non-ICI group at four time points before PSM**

| Time-points | ICI group | |  | Non-ICI group | |
| --- | --- | --- | --- | --- | --- |
|  | CAC volume (IQR) | CAC score (IQR) |  | CAC volume (IQR) | CAC score (IQR) |
| Baseline | 93.4 (37.8, 253.0) | 96.9 (34.6, 306.8) |  | 70.1 (27.4, 205.3) | 68.8 (25.3, 226.6) |
| 3 Months | 104.7 (36.2, 266.4) | 105.5 (34.6, 308.8) |  | 72.4 (30.2, 189.6) | 80.4 (25.3, 216.2) |
| 6 Months | 116.7 (39.1, 309.1) | 121.0 (35.0, 357.3) |  | 81.8 (31.3, 215.6) | 88.8 (31.2, 248.3) |
| 12 Months | 125.1 (46.0, 294.8) | 132.8 (42.4, 388) |  | 84.4 (33.9, 214.1) | 87.9 (35.7, 270.9) |

Abbreviations: CAC, coronary artery calcium; ICI, immune checkpoint inhibitor; IQR, interquartile range; PSM, propensity score matching.

**Table S11. CAC volume and score in the ICI and the non-ICI group at four time points after PSM**

| Time-points | ICI group | |  | Non-ICI group | |
| --- | --- | --- | --- | --- | --- |
|  | CAC volume (IQR) | CAC score (IQR) |  | CAC volume (IQR) | CAC score (IQR) |
| Baseline | 85.1 (26.6, 253.0) | 76.4 (26.8, 297.5) |  | 74.9 (27.4, 205.3) | 76.8 (23.4, 226.6) |
| 3 Months | 83.4 (31.5, 256.4) | 87.7 (30.1, 286.9) |  | 84.6 (30.0, 189.6) | 83.0 (23.3, 216.2) |
| 6 Months | 101.0 (33.0, 282.0) | 101.9 (28.9, 353.6) |  | 103.2 (27.6, 217.7) | 92.7 (23.5, 248.3) |
| 12 Months | 111.8 (37.3, 286.6) | 121.1 (37.8, 358.7) |  | 109.3 (29.7, 226.3) | 98.7 (30.9, 270.9) |

Abbreviations: CAC, coronary artery calcium; ICI, immune checkpoint inhibitor; IQR, interquartile range; PSM, propensity score matching.

**Table S12. Progression of CAC volume and score Between the ICI and the Non-ICI Group at 12 Months before PSM**

|  | ICI group (IQR) | Non-ICI group (IQR) | *P* values* |
| --- | --- | --- | --- |
| Absolute volume progression (mm^3^) | 29.2 (7.6, 53.7) | 10.1 (3.2, 27.0) | <0.001 |
| Absolute score progression | 34.3 (7.4, 74.9) | 12.8 (2.8, 31.1) | <0.001 |
| Relative volume progression (%) | 27.4 (13.3, 48.4) | 20.1 (5.8, 35.0) | 0.014 |
| Relative score progression (%) | 30.6 (14.2, 48.4) | 21.2 (6.1, 43.0) | 0.016 |

Abbreviations: CAC, coronary artery calcium; ICI, immune checkpoint inhibitor; IQR, interquartile range; PSM, propensity score matching.

*Wilcoxon rank sum test comparing progression of CAC volume and score between the ICI group with the non-ICI group.

**Table S13. Number of patients with different CAC score grade in the ICI and the Non-ICI group before and after PSM**

|  | Group | Before PSM | | | |  | After PSM | | | |
| --- | --- | --- | --- | --- | --- | --- | --- | --- | --- | --- |
|  |  | Baseline | 3 Months | 6 Months | 12 Months |  | Baseline | 3 Months | 6 Months | 12 Months |
| Mild calcification | ICI | 58 | 56 | 50 | 44 |  | 43 | 41 | 37 | 32 |
|  | Non-ICI | 76 | 76 | 71 | 70 |  | 42 | 42 | 39 | 39 |
| Moderate calcification | ICI | 33 | 36 | 39 | 42 |  | 20 | 23 | 24 | 27 |
|  | Non-ICI | 37 | 36 | 40 | 41 |  | 25 | 23 | 26 | 26 |
| Severe calcification | ICI | 22 | 21 | 24 | 27 |  | 12 | 11 | 14 | 16 |
|  | Non-ICI | 20 | 22 | 22 | 22 |  | 8 | 10 | 10 | 10 |

Abbreviations: CAC, coronary artery calcium; ICI, immune checkpoint inhibitor; PSM, propensity score matching.

Mild calcification: CAC score=1-99; Moderate calcification: CAC score=100-400; Severe calcification: CAC score >400.

**Table S14. CAC score grade progression in the ICI and the Non-ICI group before and after PSM**

| Time-points | Before PSM | | |  | After PSM | | |
| --- | --- | --- | --- | --- | --- | --- | --- |
|  | ICI group | Non-ICI group | *P* values* |  | ICI group | Non-ICI group | *P* values* |
| 3 Months progression | 3 (2.7%) | 6 (4.5%) | 0.513 |  | 3 (4.0%) | 5 (6.7%) | 0.719 |
| 6 Months progression | 10 (8.8%) | 8 (6.0%) | 0.395 |  | 8 (10.7%) | 6 (8.0%) | 0.575 |
| 12 Months progression | 19 (16.8%) | 9 (6.8%) | 0.013 |  | 15 (20.0%) | 6 (8.0%) | 0.032 |

Abbreviations: CAC, coronary artery calcium; ICI, immune checkpoint inhibitor; PSM, propensity score matching.

*Chi-square test comparing progression of three time points in CAC score grade between ICI group with non-ICI group.

**Table S15. Multiple linear regression analysis for associations of ICIs with absolute volume progression, absolute score progression, relative volume progression and relative score progression of CAC at 12 months before PSM**

|  | Model 1 | |  | Model 2 | |  | Model 3 | |
| --- | --- | --- | --- | --- | --- | --- | --- | --- |
|  | Beta (SE) | *P* value |  | Beta (SE) | *P* value |  | Beta (SE) | *P* value |
| Absolute volume progression | 0.135 (0.066) | 0.042 |  | 0.119 (0.068) | 0.078 |  | 0.127 (0.052) | 0.015 |
| Absolute score progression | 0.153 (0.067) | 0.022 |  | 0.149 (0.069) | 0.030 |  | 0.130 (0.046) | 0.005 |
| Relative volume progression | 0.202 (0.069) | 0.003 |  | 0.188 (0.067) | 0.005 |  | 0.201 (0.065) | 0.002 |
| Relative score progression | 0.167 (0.068) | 0.014 |  | 0.176 (0.071) | 0.013 |  | 0.171 (0.068) | 0.012 |

Abbreviations: ICI, immune checkpoint inhibitor; CAC, coronary artery calcium; SE, standard error; PSM, propensity score matching.

Model 1 is adjusted for sex, age and history of cardiovascular disease.

Model 2 is adjusted for body mass index, hypertension, diabetes, smoking index, hyperlipidemia, chest radiation therapy, statins, aspirin, hemoglobin, neutrophil to lymphocyte ratio and platelet count to lymphocyte ratio in addition to the variables in the model 1.

Model 3 is adjusted for baseline coronary artery calcium volume and score, in addition to the variables in the model 2.


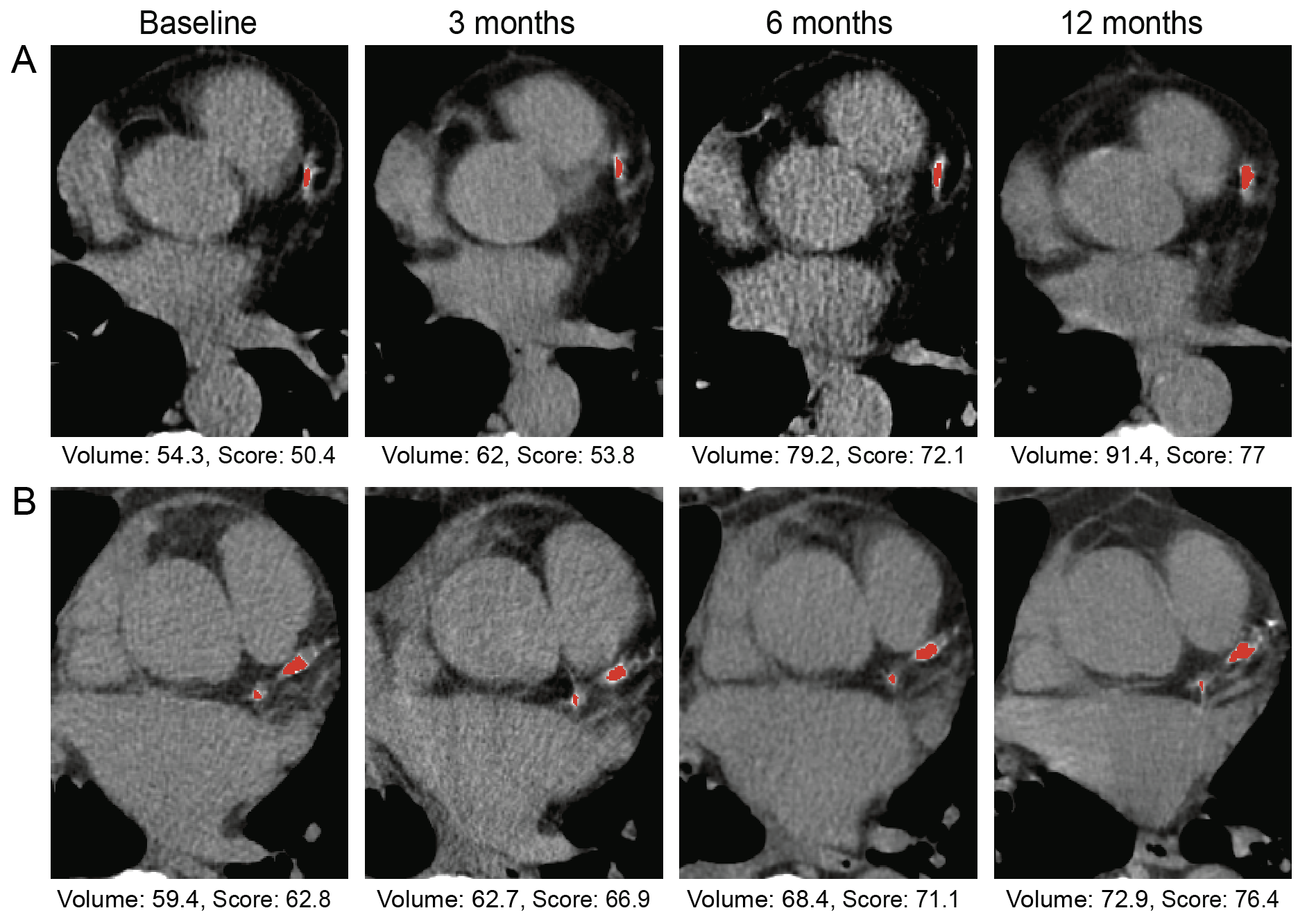


**Fig. S1.** Representative axial CT images show the progression of CAC volume and score in patients with and without ICI

therapy. Quantitative CT of coronary calcified plaque (red) at baseline and at 3 months, 6 months, and 12 months after treatment.

**(A)** an adult patient treatment with PD-1 antibody, and **(B)** an adult patient without ICI therapy. Abbreviations: CAC, coronary

artery calcium; ICI, immune checkpoint inhibitor; PD-1, programmed cell death ligand 1.


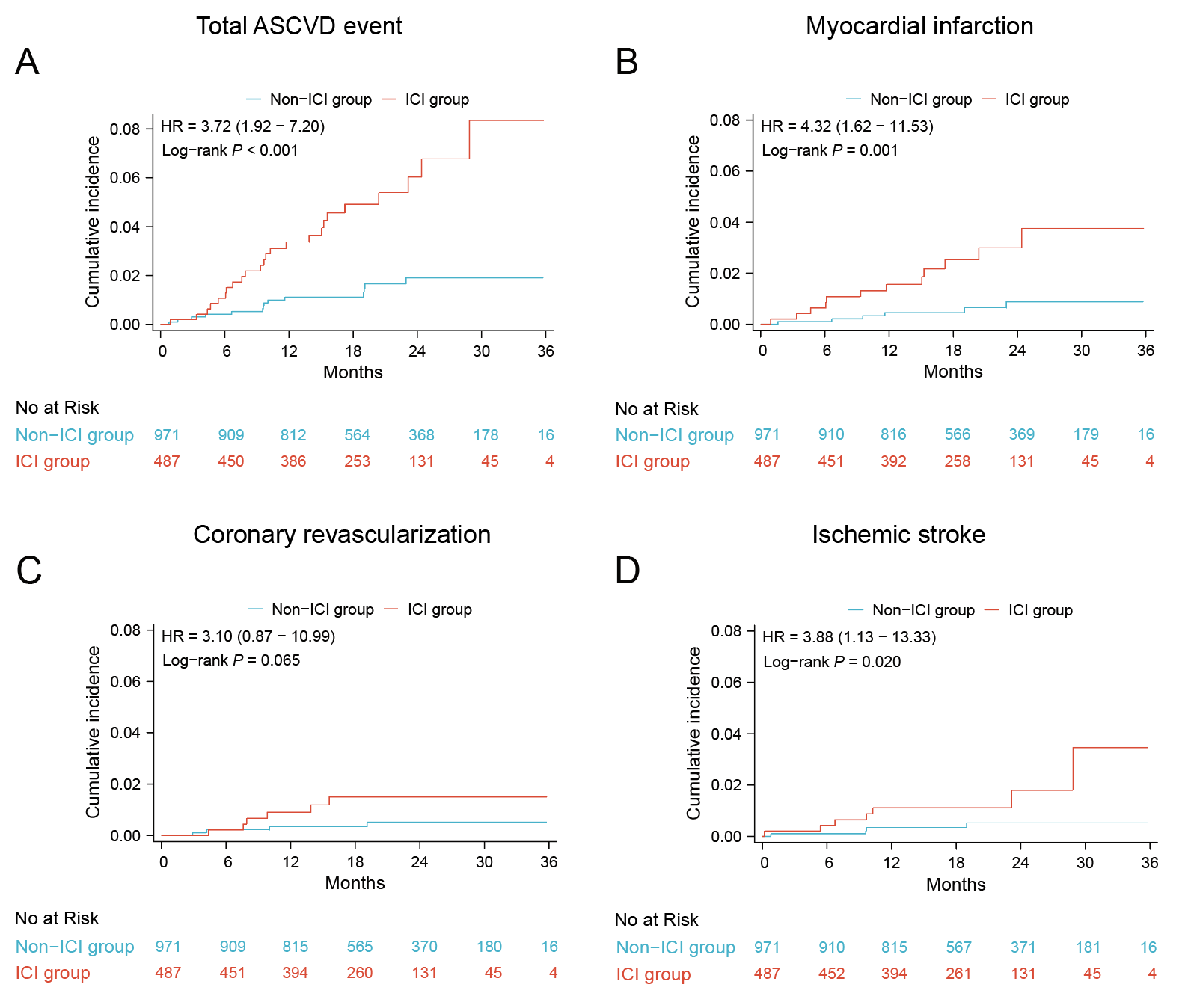


**Fig. S2.** Kaplan–Meier curves of the cumulative incidence of the ASCVD events between the ICI and the non-ICI group before PSM. **(A)** Cumulative incidence for the total ASCVD event. **(B-D)** Cumulative incidence for the individual components of the ASCVD events. Abbreviations: HR, hazard ratio; ASCVD, atherosclerotic cardiovascular disease; ICI, immune checkpoint inhibitor; PSM, propensity score matching.


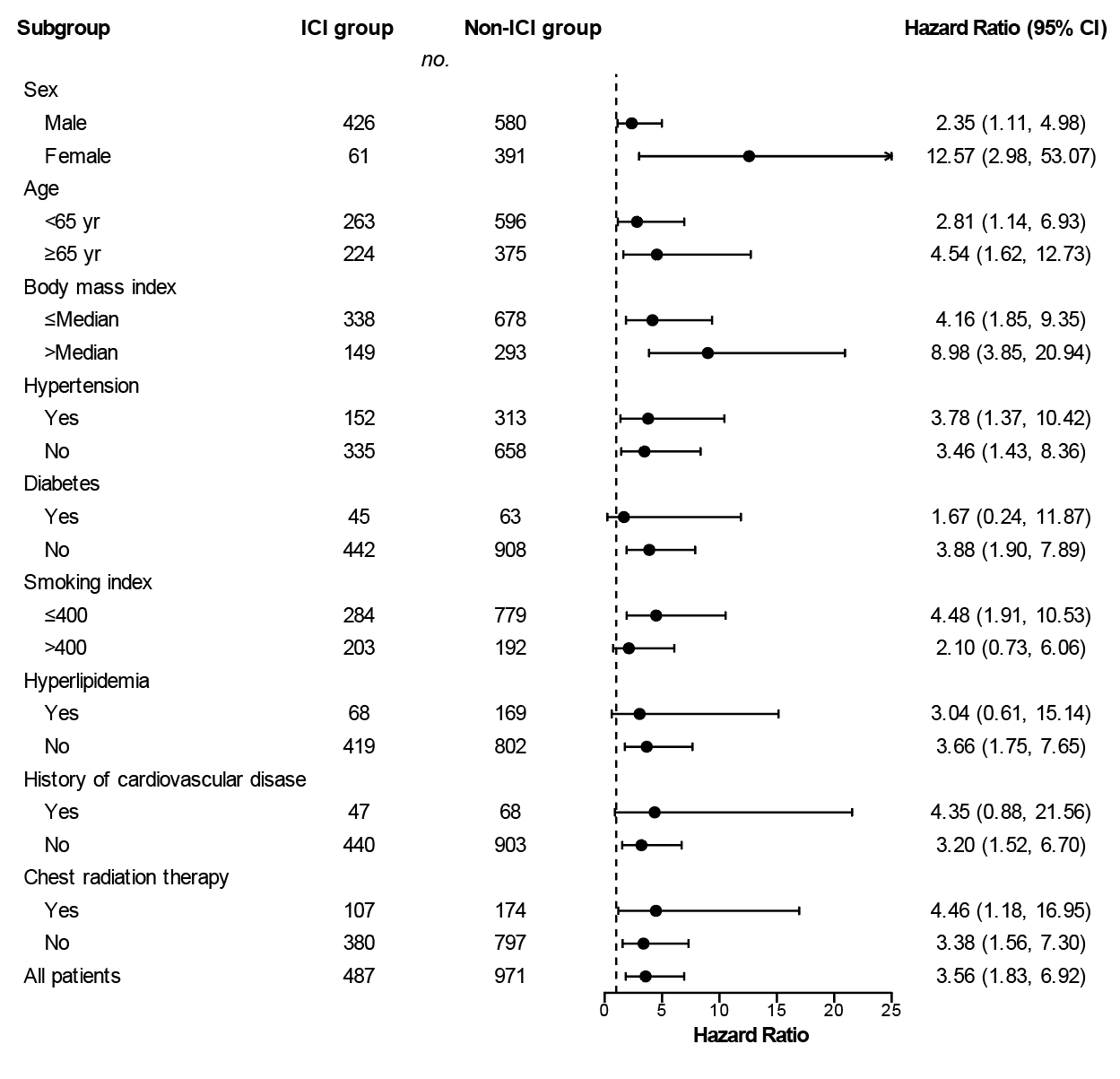


**Fig. S3.** Forest plot of the hazard ratios for the total ASCVD event stratified by different subgroups before PSM. Hazard

ratios were derived from univariate Cox model for each subgroup. Dashed line indicates HR of 1. Abbreviations: ASCVD,

atherosclerotic cardiovascular disease; PSM, propensity score matching.


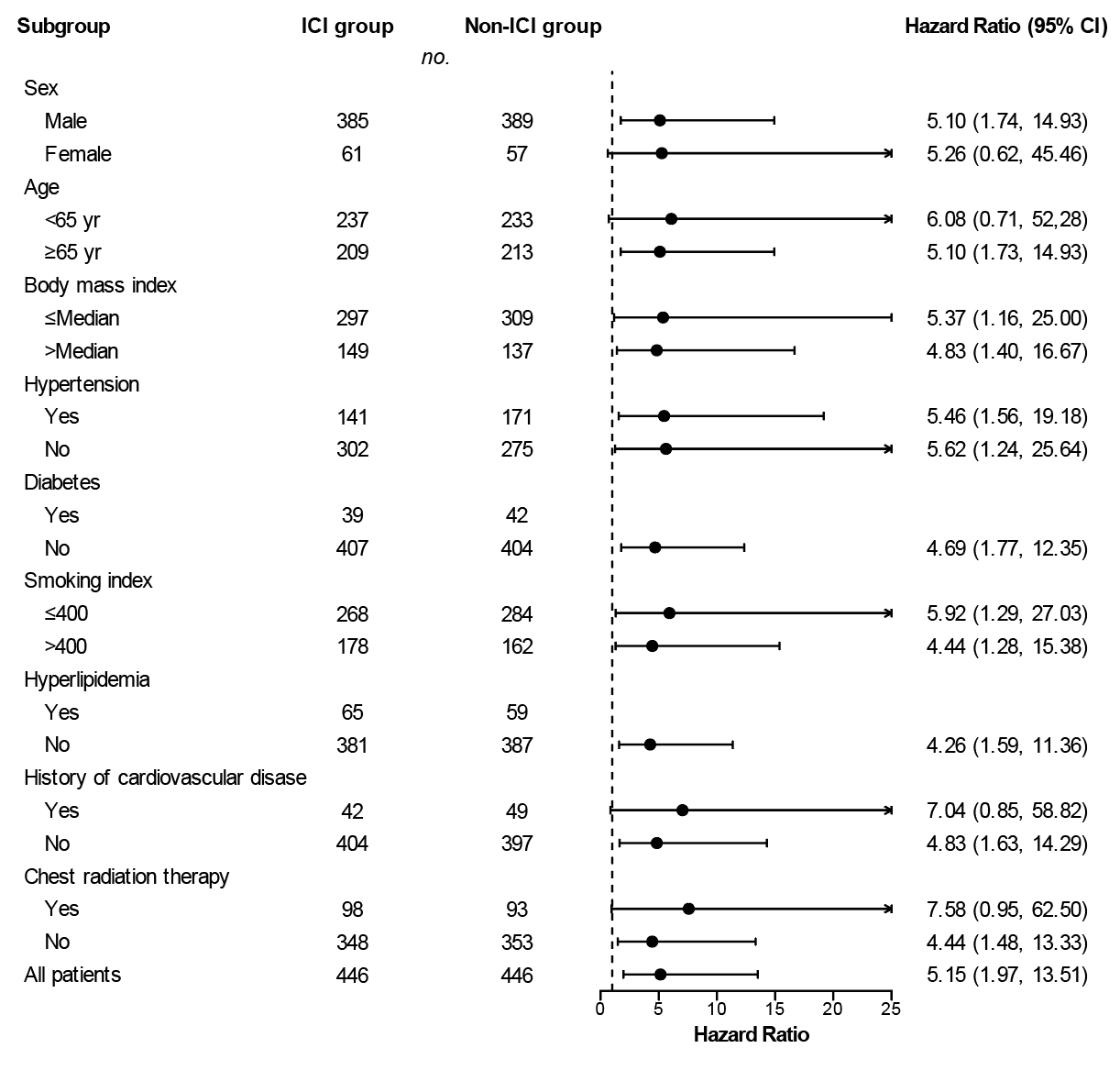


**Fig. S4.** Forest plot of the hazard ratios for the total ASCVD event stratified by different subgroups after PSM. Hazard

ratios were derived from univariate Cox model for each subgroup. Dashed line indicates HR of 1. Abbreviations: ASCVD,

atherosclerotic cardiovascular disease; PSM, propensity score matching.


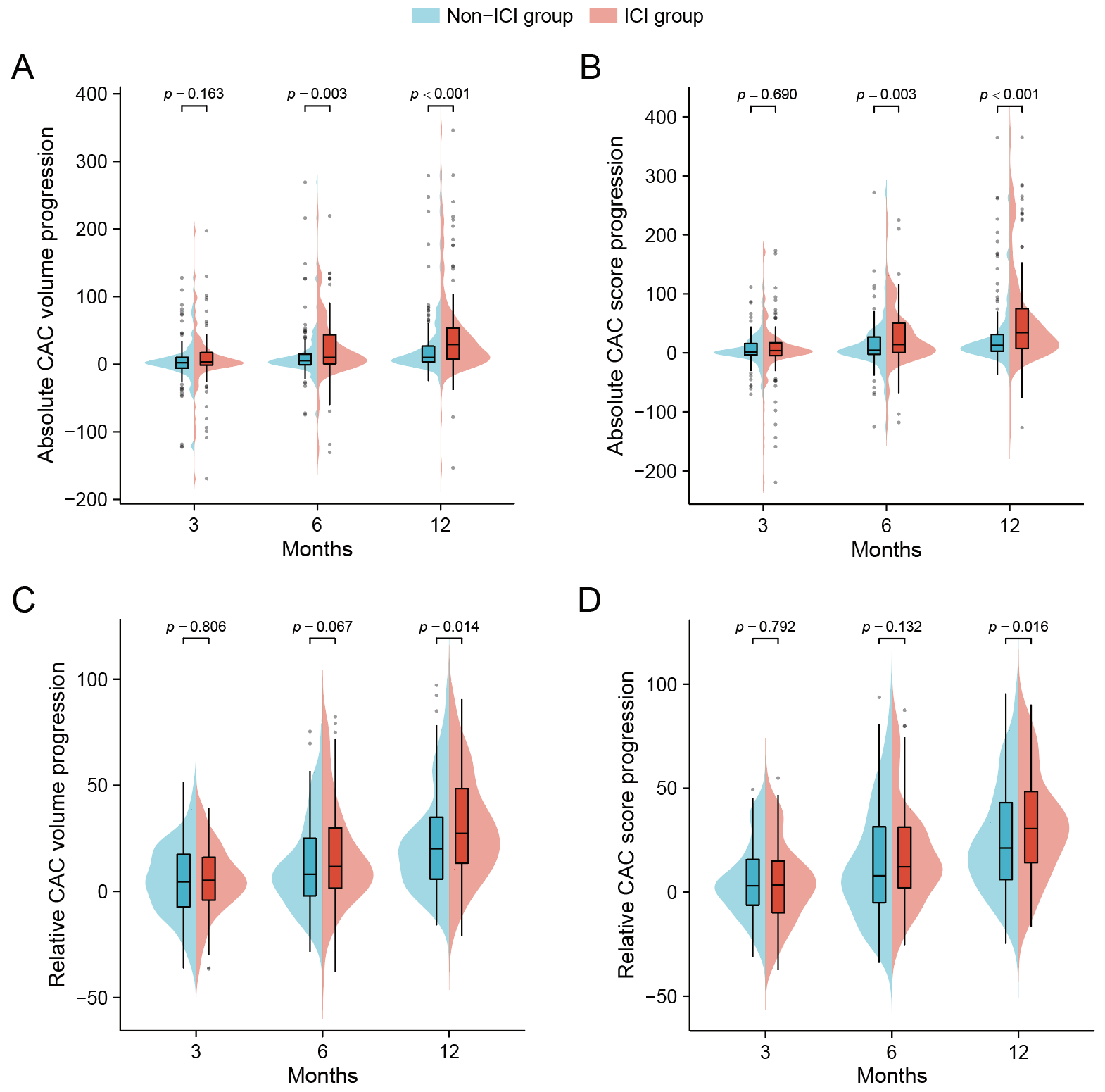


**Fig. S5.** Bean plots show time-course progression of the CAC volume and score between the ICI and the non-ICI group before PSM. **(A)** Absolute

CAC volume progression. **(B)** Absolute CAC score progression. **(C)** Relative CAC volume progression. **(D)** Relative CAC score progression. Abbreviations: CAC, coronary artery calcium; ICI, immune checkpoint inhibitor; PSM, propensity score matching.


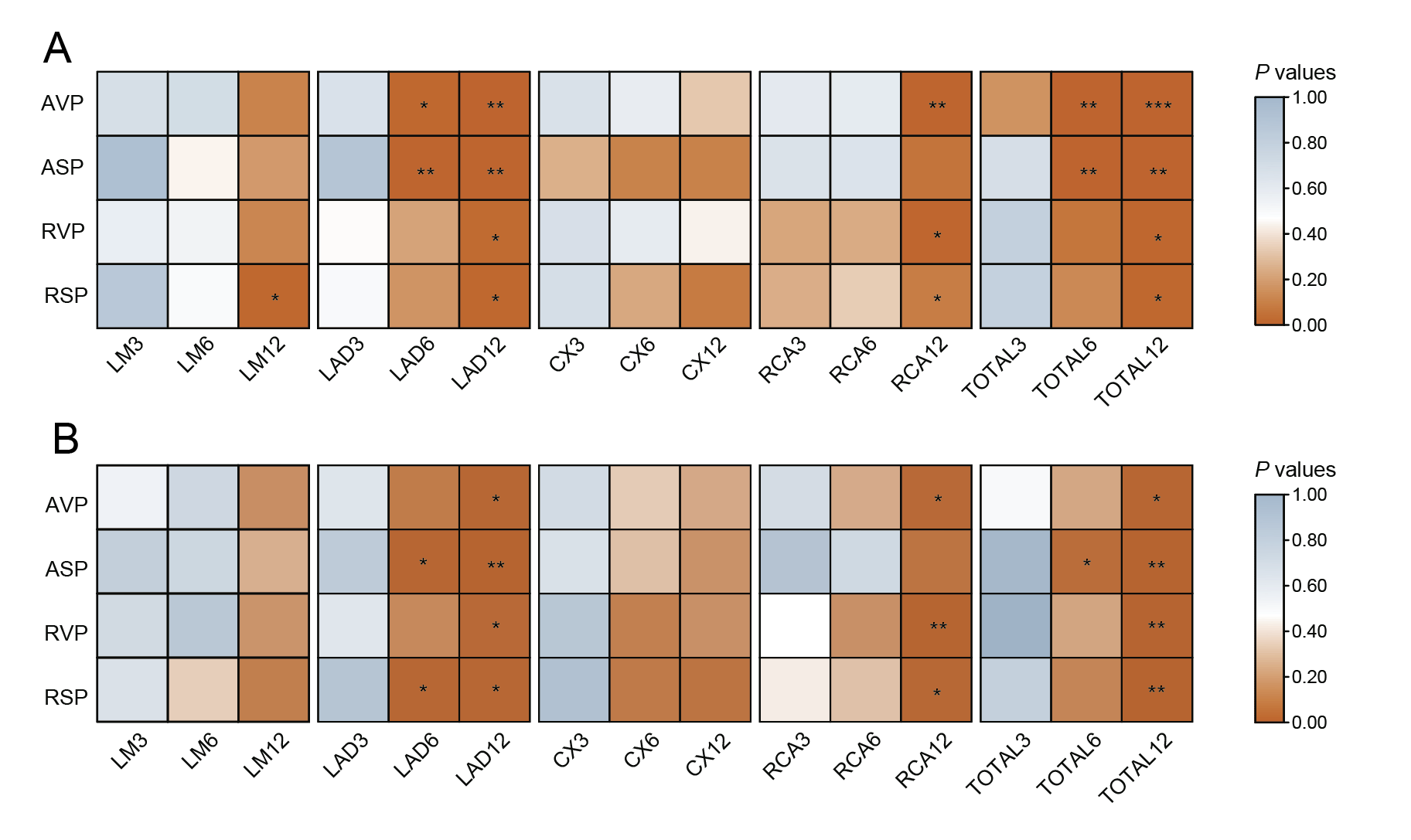


**Fig. S6.** Heatmap of CAC progression in each segment of the coronary artery before **(A)** and after **(B)** PSM. Abbreviations: CAC,

coronary artery calcium; PSM, propensity score matching; AVP, absolute volume progression; ASP, absolute score progression; RVP,

relative volume progression; RSP, relative score progression; LM, left main trunk; LAD, left anterior descending artery; CX, circumflex;

and RCA, right coronary artery.


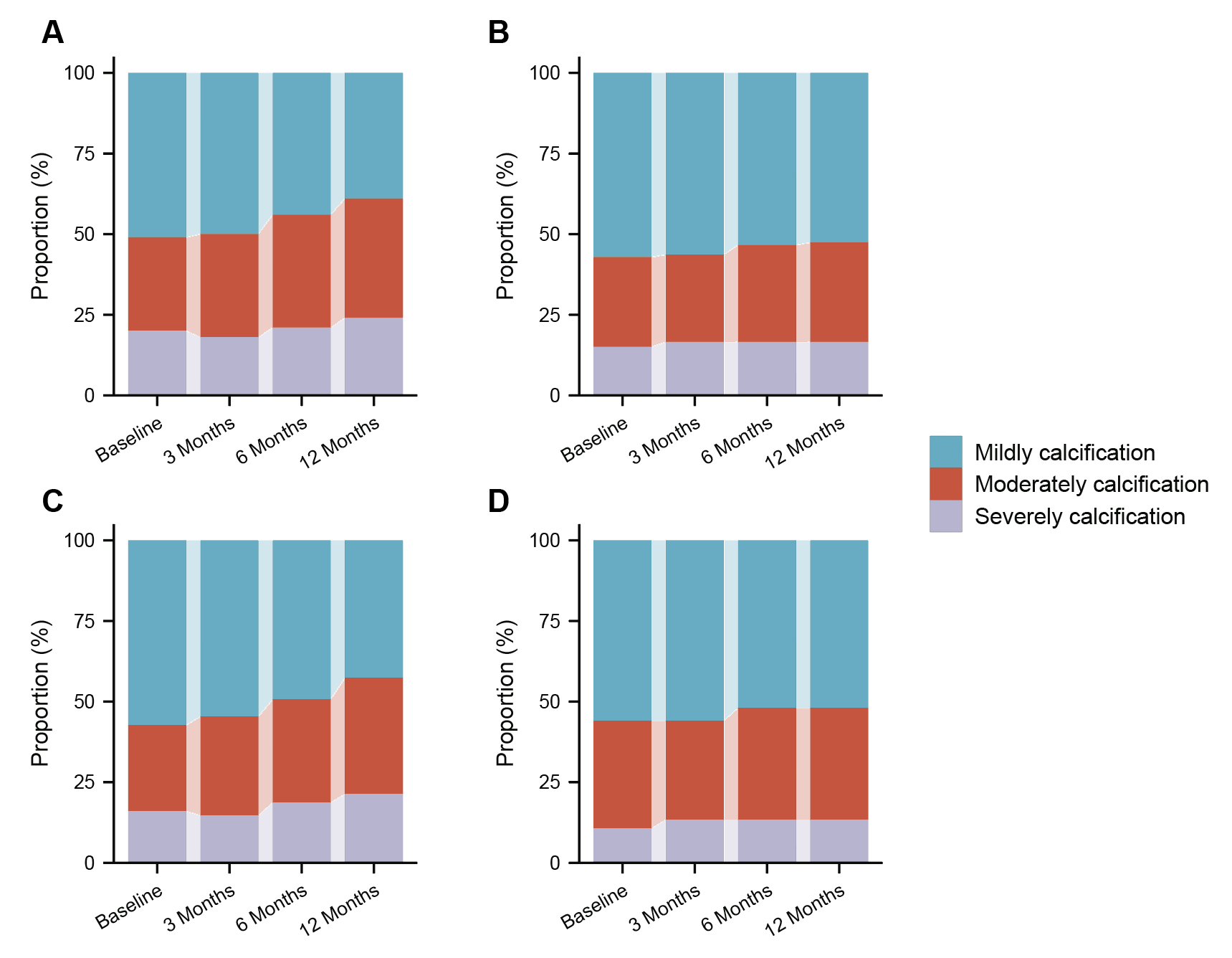


**Fig. S7.** Stacked bar graphs of CAC grade progression in ICI **(A)** and non-ICI **(B)** group before PSM and in ICI **(C)** and non-ICI **(D)**

group after PSM. Stacked bars of three different colors represent the proportion of different degrees of calcification in each group.

Abbreviations: CAC, coronary artery calcium; ICI, immune checkpoint inhibitor; PSM, propensity score matching.


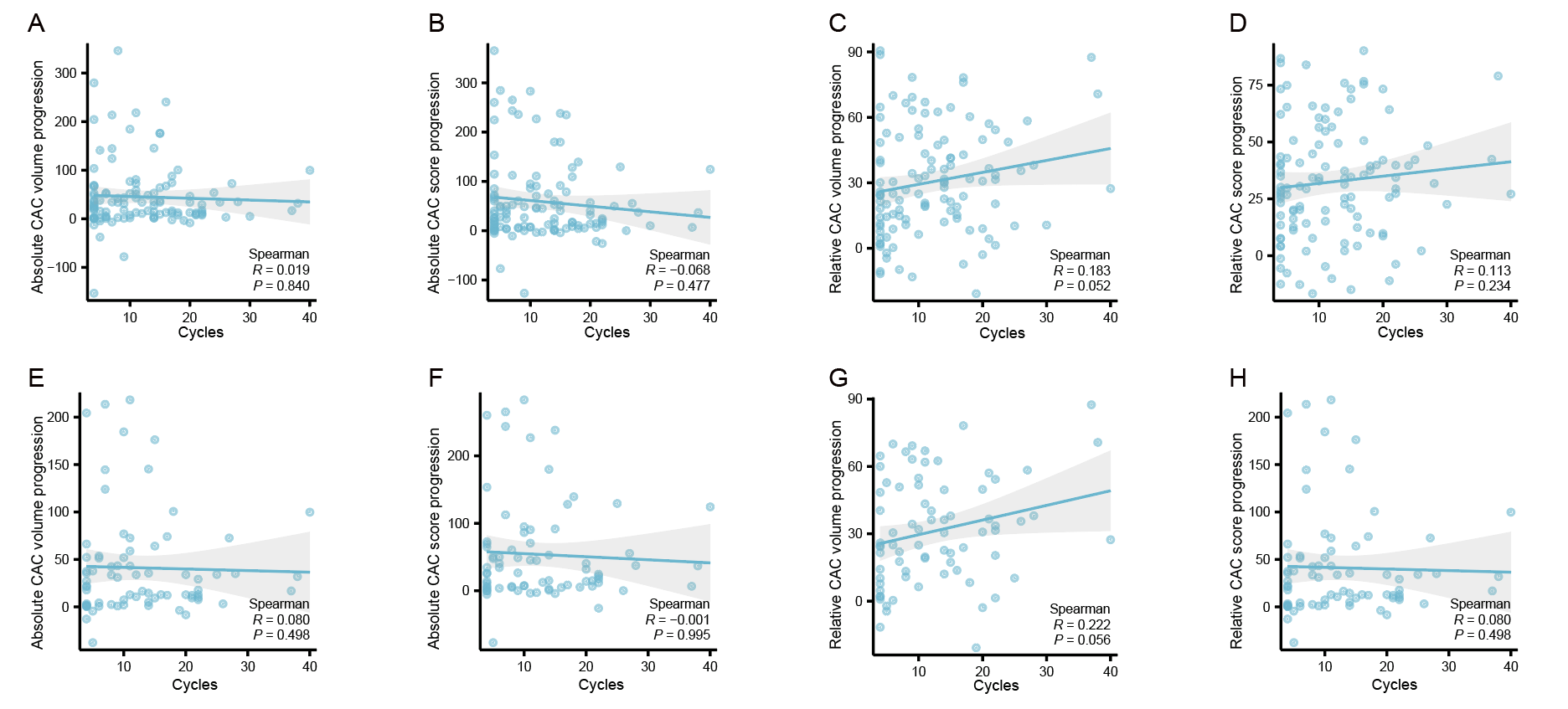


**Fig. S8.** Scatter plots of cycles of ICI versus absolute volume progression, absolute score progression, relative volume progression and relative

score progression of CAC at 12 months before **(A-D)** and after **(E-H)** PSM. Abbreviations: ICI, immune checkpoint inhibitor; CAC, coronary artery

calcium; PSM, propensity score matching.
